# A pan-cancer analysis reveals the oncogenic and immunological role of insulin-like growth factor 2 mRNA-binding protein family members

**DOI:** 10.1007/s12672-025-02077-2

**Published:** 2025-03-15

**Authors:** Fuling Zeng, Liuyan Chen, Jing Li, Wenna Yu, Niya Sa, Keke Zhang, Chen Qu, Daolin Wen

**Affiliations:** 1 Department of Laboratory Medicine, Shenzhen Guangming District People’s Hospital, Shenzhen, 518000 Guangdong China; 2 Department of Pathophysiology, School of Medicine, Jinan University, Guangzhou, 510632 Guangdong China; 3 College of Pharmacy, Jinan University, Guangzhou, 510632 Guangdong China

**Keywords:** m^6^A, IGF2BPs, Pan-cancer, Prognosis, Immunotherapy

## Abstract

**Purpose:**

To investigate the expression and clinical significance of insulin-like growth factor 2 mRNA-binding protein family members (IGF2BPs) in pan-cancer and evaluate their potential as targets for tumor immunotherapy.

**Methods:**

Based on data from the cancer genome atlas (TCGA) database, pan-cancer analysis was conducted to examine the clinical significance of IGF2BPs expression in twenty-two tumors.

**Results:**

Differential expression analysis showed high expression of IGF2BPs in most tumor tissues. Survival and mutation analyses suggested that the overexpression of IGF2BPs was associated with poor prognosis and mutation status of certain tumors. Methylation analysis revealed the methylation levels of IGF2BP1/2/3 in certain tumors were intricately linked to their mRNA expression, patient prognosis, and immune cell infiltration. Enrichment analysis indicated that abnormal expression of IGF2BPs was associated with various common tumor-related pathways in different tumors, including AMPK, Hippo, PI3K-Akt, EMT, and p53. In addition, immune correlation analysis revealed that IGF2BPs were closely related to immunotherapy-related indicators (immune cell infiltration, major histocompatibility complex (MHC), immune checkpoints, tumor mutation burden (TMB), and microsatellite instability (MSI)) in some tumors. Drug sensitivity analysis indicated that IGF2BPs were sensitive to some common chemotherapeutic drugs (alvocidib, dasatinib, trametinib, and selumetinib).

**Conclusion:**

IGF2BPs exhibit significantly high expression in most tumors and are associated with prognosis, pathological stage, mutational status, methylation levels, and the relevant indicators of immunotherapy sensitivity in multiple tumors. Moreover, IGF2BPs may play an oncogenic role by activating common signaling pathways. Therefore, IGF2BPs may be potential prognostic markers for tumor therapy and targets for immunotherapy and drug therapy.

**Supplementary Information:**

The online version contains supplementary material available at 10.1007/s12672-025-02077-2.

## Introduction

Recent studies have shown that m6A modification plays a crucial role in the occurrence, development, metabolism, drug resistance, and immune evasion of tumors [1–3]. The biological function of m6A modification is influenced by m6A reader proteins, among which the IGF2BPs (insulin-like growth factor 2 mRNA-binding proteins) gene family, serving as m6A readers, can recognize and interact with m6A-modified mRNAs to regulate post-transcriptional modifications [4, 5]. The IGF2BPs gene family is a conserved family of m6A reader proteins, including members such as IGF2BP1, IGF2BP2, and IGF2BP3 [6]. Their amino acid sequence homology exceeds 56%, indicating that they have similar structures and have similar biological functions [7]. The structure of IGF2BPs contains two RNA recognition motifs (RRMs) and four K homologous domains (KH), where the RRM domains primarily bind to target mRNA, forming stable ribonucleoprotein particles (RNPs) [8–10], while KH domains are responsible for m6A recognition and binding [11–13]. Therefore, IGF2BPs can regulate mRNA stability, translation, localization, and degradation modified by m6A through these structures, subsequently affecting the expression of related genes. IGF2BPs play a crucial role in important biological processes such as embryonic development, organ and nervous system development, cell self-renewal and differentiation, energy metabolism, and regulation of the immune response [14].

Importantly, increasing evidence suggests that IGF2BPs can bind target mRNAs (mainly oncogenic mRNAs) to increase the stability of oncogenic mRNAs (such as MYC), thereby regulating cell proliferation, migration, and invasion, promoting tumor progression [15, 16]. Other m6A readers, like FTO and ALKBH5, primarily influence mRNA stability through demethylation processes, with their functions centering more on the dynamic regulation of global m6A levels [17, 18]. Additionally, IGF2BP2 is widely expressed in both normal and tumor tissues, while IGF2BP1 and IGF2BP3 are overexpressed in many malignant tumors and are considered oncofetal proteins [19]. The abnormal high expression of IGF2BPs in various tumors, including lung cancer [20], liver cancer [21], cervical cancer [22], head and neck squamous cell carcinoma [23], enhances the proliferation, migration, and invasion of tumor cells, promoting tumor progression and strongly correlating with poor prognosis for patients. In contrast, other m6A readers, such as YTHDF1 and YTHDF2, may display expression patterns and functions that are more dependent on specific cancer types and do not consistently show carcinogenic characteristics [24]. Despite strong evidence supporting their role as oncogenes and influencing tumor cells aspects such as self-renewal, apoptosis, metabolic reprogramming, and immune evasion [25, 26], recent studies have found that IGF2BP1 plays a tumor-suppressive role in various tumors. For example, high expression of IGF2BP1 has been found to decrease the growth and invasiveness of breast cancer [27]. In gallbladder cancer tissues, IGF2BP1 expression is lower than in normal tissues, and low expression predicts a good prognosis for patients [28]. Additionally, knocking down IGF2BP1 in colonic stromal cells promotes the tumor microenvironment and histological grading [29, 30]. These findings suggest that the role of IGF2BPs may vary across different tumors.

In addition, several studies have indicated that IGF2BPs participate in the tumor immune process, leading to immune suppression and evasion, thereby promoting tumor progression [31]. Specifically, Previous studies have shown that high expression of IGF2BP1 can reduce the infiltration of immune cells such as T cells and macrophages in tumors, thereby promoting the expression of PD-L1 [32]. IGF2BP2 directly binds to mRNA associated with PD-L1, promoting its expression and leading to the formation of the immune-suppressive microenvironment in pancreatic cancer [33]. Furthermore, IGF2BP2 is involved in immune responses in colorectal cancer and can induce macrophages from M1 type to M2 type (the latter having pro-tumor activity) [34] in ovarian cancer. These findings suggest that the IGF2BPs are closely related to tumor immunity and play a crucial role in regulating the tumor immune microenvironment, immune checkpoints, and immune cell functions, and highlighting their potential as targets for immunotherapy.

Therefore, to further investigate their role in various types of tumors, we conducted a comprehensive pan-cancer analysis of the IGF2BPs gene family to enhance our understanding of their involvement in cancer development and immune regulation, and explore their potential as biomarkers for tumor prognosis assessment and targets for immunotherapy.

## Materials and methods

### Data source

In this study, we focused on tumor types that had both adjacent normal and tumor tissue data to explore the potential role and mechanisms of IGF2BPs in cancer development. To ensure the reliability and accuracy of our analysis, we excluded tumor types that lacked adjacent normal tissue data, had an insufficient sample size for statistical analysis, and contained significant missing or inconsistent data. Ultimately, we identified 22 tumor types for our research. We downloaded the complete RNA-seq data, clinical information data, mutation data and methylation data of 22 tumors in the TCGA database (https://portal.gdc.cancer.gov/) from the UCSC Xena database (https://xenabrowser.net/datapages/). The tumor types included Bladder Urothelial Carcinoma (BLCA), Breast invasive carcinoma (BRCA), Cervical squamous cell carcinoma and endocervical adenocarcinoma (CESC), Cholangio carcinoma (CHOL), Colon adenocarcinoma (COAD), Esophageal carcinoma (ESCA), Glioblastoma multiforme (GBM), Head and Neck squamous cell carcinoma (HNSC), Kidney Chromophobe (KICH), Kidney renal clear cell carcinoma (KIRC), Kidney renal papillary cell carcinoma (KIRP), Liver hepatocellular carcinoma (LIHC), Lung adenocarcinoma (LUAD), Lung squamous cell carcinoma (LUSC), Pancreatic adenocarcinoma (PAAD), Pheochromocytoma and Paraganglioma (PCPG), Prostate adenocarcinoma (PRAD), Rectum adenocarcinoma (READ), Skin Cutaneous Melanoma (SKCM), Stomach adenocarcinoma (STAD), Thyroid carcinoma (THCA), and Uterine Corpus Endometrial Carcinoma (UCEC). Furthermore, we downloaded the expression matrix and clinical information of the GSE176307, GSE103668, GSE165252, GSE53127, and GSE78220 datasets from the GEO database (https://www.ncbi.nlm.nih.gov/gds/). Additionally, we downloaded the expression matrix and clinical information of the ERP105482, SRP094781, SRP150548, SRP155030, SRP230414, and SRP302761 datasets from the SRA database (https://sra-explorer.info/).

### Differential expression analysis

By normalizing mRNA expression matrices using TPM or FPKM across different tumors, we performed a t-test to the IGF2BP1/2/3 mRNA expression levels in different tumor tissues and adjacent normal tissues (the two sets of data are normally distributed and have equal variances) and obtained the significance levels (***P < 0.001, **P < 0.01, and *P < 0.05). We then calculated the average expression levels of IGF2BP1/2/3 in different tumor and adjacent normal tissues. The R package “pheatmap” was used to draw the mRNA expression heatmap of IGF2BP1/2/3 in different tumor and adjacent normal tissues. In addition, we combined clinical pathological staging information with mRNA expression data and used t-test to analyze the differences in IGF2BP1/2/3 expression in different pathological staging subgroups (stage I and II groups vs. stage III and IV groups) for each tumor type, and obtained significant levels (***P < 0.001, **P < 0.01, and *P < 0.05). Finally, we used the R package “ggplot2” to visualize the results and create heatmaps and violin plots.

### Survival analysis

By merging clinical survival data (Overall Survival) with mRNA expression data, we utilized the R package “Survival” to establish a Cox proportional hazard model. This model allowed us to analyze the hazard ratio (HR) of the IGF2BP1/2/3 subgroups (the high expression group and the low expression group) in different tumors. Because PRAD patients usually require a longer follow-up time to obtain reliable overall survival data, relying on OS as the primary endpoint may lead to bias and uncertainty in the analysis results. Therefore, we used PRAD's PFS data. The log-rank test was used to analyze whether there were differences in overall survival between the two subgroups (***P < 0.001, **P < 0.01, and *P < 0.05), and the R package “ggplot2” was used for the heatmap visualization. Finally, the R package “Survival” was used to create the Kaplan–Meier survival curve of the IGF2BP1/2/3 subgroups (the high expression group and the low expression group).

### Mutation analysis

Utilizing the cBioPortal database (http://www.cbioportal.org/), we obtained genetic variation information of IGF2BP1/2/3 across various tumor types to investigate the influence of these gene mutations on patient prognosis. We used the R packages “ggplot2” and “ggpubr” to create CNV, SNV distribution maps, heatmaps and pie charts to illustrate the comprehensive genetic alteration status of IGF2BP1/2/3. Next, we merged the CNV data with clinical survival data and mRNA expression data. We used the R package “Survival” to fit survival time and survival status within the genetic alteration and unalteration groups. We performed the log-rank test to assess survival differences between genetic alteration group and unalteration group. Finally, the Spearman correlation analysis was performed to evaluate the correlation between gene mRNA expression and CNV.

### Methylation analysis

Through methylation and clinical data in diverse tumors within the TCGA dataset, we analyzed and compared the methylation levels of IGF2BP1/2/3 between different tumor tissues and adjacent normal tissues. We conducted the Spearman correlation analysis to investigate the relationships between mRNA expression levels of IGF2BP1/2/3 and their methylation levels (Correlation coefficient range: − 1 ~ 1). We performed the t-test on the calculated Spearman correlation coefficient to determine whether the correlation was significant (***P < 0.001, **P < 0.01, and *P < 0.05).

We combined the methylation data with clinical survival data and categorized the tumor samples into hypermethylation and hypomethylation groups based on the median methylation level. We employed the R package “Survival” to establish a Cox proportional hazards model to calculate the hazard ratio (HR) of the high methylation group compared to the low methylation group. The log-rank test was used to analyze the differences in overall survival (OS), disease-free survival (DFS), and disease-specific survival (DSS) of the two subgroups. When the HR between the hypermethylation group and the hypomethylation group is greater than 1, we concluded that the group with a higher degree of methylation had a higher risk of death, while the group with a lower degree of methylation had a lower risk of death.

Moreover, we utilized the R package “Xcell” to analyze the immune cell infiltration of different tumor types (B cells, CD4 + T, CD8 + T, DC, macrophages, and neutrophils). We then conducted Spearman correlation analyses to explore the correlation between the methylation levels of IGF2BP1/2/3 and immune cell infiltration. The calculated Spearman correlation coefficients were then subjected to the t-test to determine whether the correlation was significant (***P < 0.001, **P < 0.01, *P < 0.05).

### Functional enrichment analysis.

We utilized the STRING database (https://string-db.org/) to acquire the interacting proteins of IGF2BP1/2/3, setting a threshold of interaction coefficient > 0.2 to extract the top 500 interacting proteins. The interaction coefficients of these proteins were determined based on various methods, nincluding Textmining, Experiments, Databases, Coexpression, Neighborhood, and Gene Fusion and Cooccurrence. We determined the common interacting protein-coding genes of IGF2BP1/2/3 by taking the intersection. Additionally, we employed the GEPIA2 database (http://gepia2.cancer-pku.cn/) to obtain the top 500 co-expressed genes of IGF2BP1/2/3. Furthermore, we performed the Gene Ontology (GO) and Kyoto Encyclopedia of Genes and Genomes (KEGG) enrichment analysis. We used the Benjamini–Hochberg statistical method correction to compare significant differences between the input gene set and known functional annotations or pathway data (***P < 0.001, **P < 0.01, *P < 0.05). Finally, we analyzed the correlation between the expression levels of IGF2BP1/2/3 and the activation/inhibition status of various tumor-related pathways.

### Immune correlation analysis.

The correlation between the expression levels of IGF2BP1/2/3 and immune infiltration was analyzed using the R package “Estimate”. The calculated Spearman correlation coefficient was then subjected to the t-test to determine whether the correlation was significant (***P < 0.001, **P < 0.01, *P < 0.05). The stromal score reflects the abundance of infiltrating stromal cells in tumor tissue, while the immune score represents the status of infiltrating immune cells within tumor tissue. Additionally, the estimated score is used to assess tumor purity.

Furthermore, the R package "Xcell" was utilized to analyze the correlation of IGF2BP1/2/3 expression levels with immune cell infiltration, immune checkpoints, TMB (tumor mutational burden) and MSI (microsatellite instability) in different tumors. The Spearman correlation coefficient was then subjected to the t-test to determine whether the correlations were significant (***P < 0.001, **P < 0.01, *P < 0.05). In addition, we assessed the association between IGF2BP1/2/3 expression and MHC molecules across different tumors using the TISIDB online database (http://cis.hku.hk/TISIDB/index.php). By merging clinical information with mRNA expression matrix data, we employed the t-test to compare the expression levels of IGF2BP1/2/3 in immune responders and non-responders, as well as to analyze the differences in IGF2BP1/2/3 expression before and after immunotherapy (***P < 0.001, **P < 0.01, *P < 0.05). After patients with tumors undergo immunotherapy, the efficacy evaluation uses solid tumor response evaluation criteria includes the following categories: complete response (CR), partial response (PR), stable disease (SD), and progressive disease (PD). Clinically, patients with complete response and partial response in efficacy evaluation are considered as immune responders, while patients with stable disease and progressive disease are considered as non-immune responders.

### Drug sensitivity analysis

Via the Genomics of Drug Sensitivity in Cancer (GDSC) (https://www.cancerrxgene.org/) and the Cancer Therapeutics Response Portal (CTRP) database (https://portals.broadinstitute.org), we merged IGF2BP1/2/3 mRNA expression data with drug sensitivity data to analyze the correlation between drug sensitivity and resistance and IGF2BP1/2/3 mRNA expression. The calculated Spearman correlation coefficients were subsequently subjected to the t-test to determine whether the correlations were significant (***P < 0.001, **P < 0.01, *P < 0.05).

### Statistical analysis

**Fig. 1:**
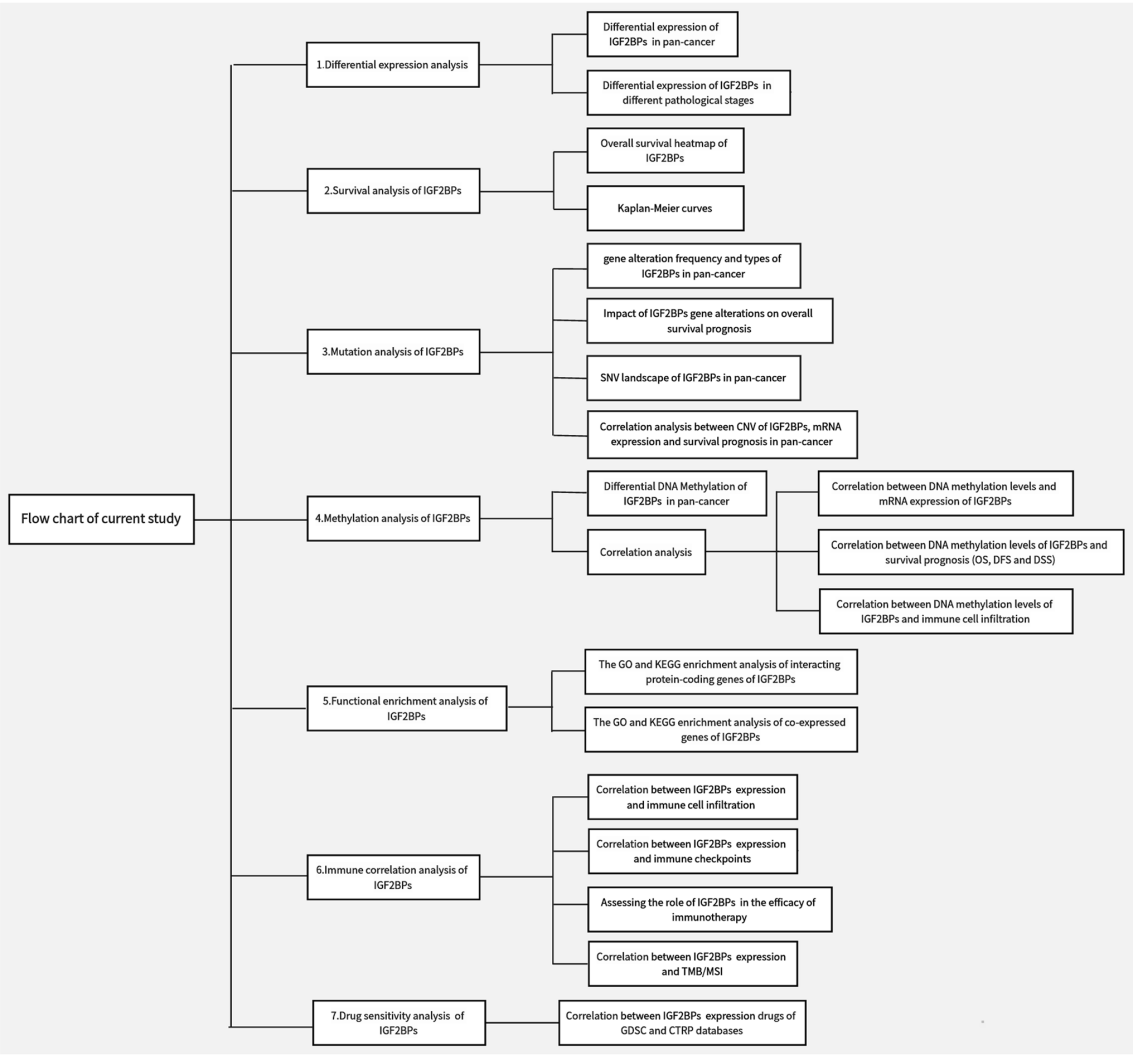
Flowchart of this study

We utilized SPSS 22.0 software and GraphPad Prism version 8.0.1 for data processing and statistical analysis. To compare groups, we employed analysis of variance (ANOVA) or independent t-test. A significance level of *P* < 0.05 was considered statistical significance (see Fig. 1).

## Results

### Differential expression of IGF2BPs gene family in different tumors

By comparing the differences in gene expression between tumor and adjacent normal tissues, we can identify potential cancer-driving genes that are highly expressed in tumors. These driver genes play a crucial role in the initiation, progression, and metastasis of tumors, contributing to revealing the molecular mechanisms of cancer.

**Fig. 2:**
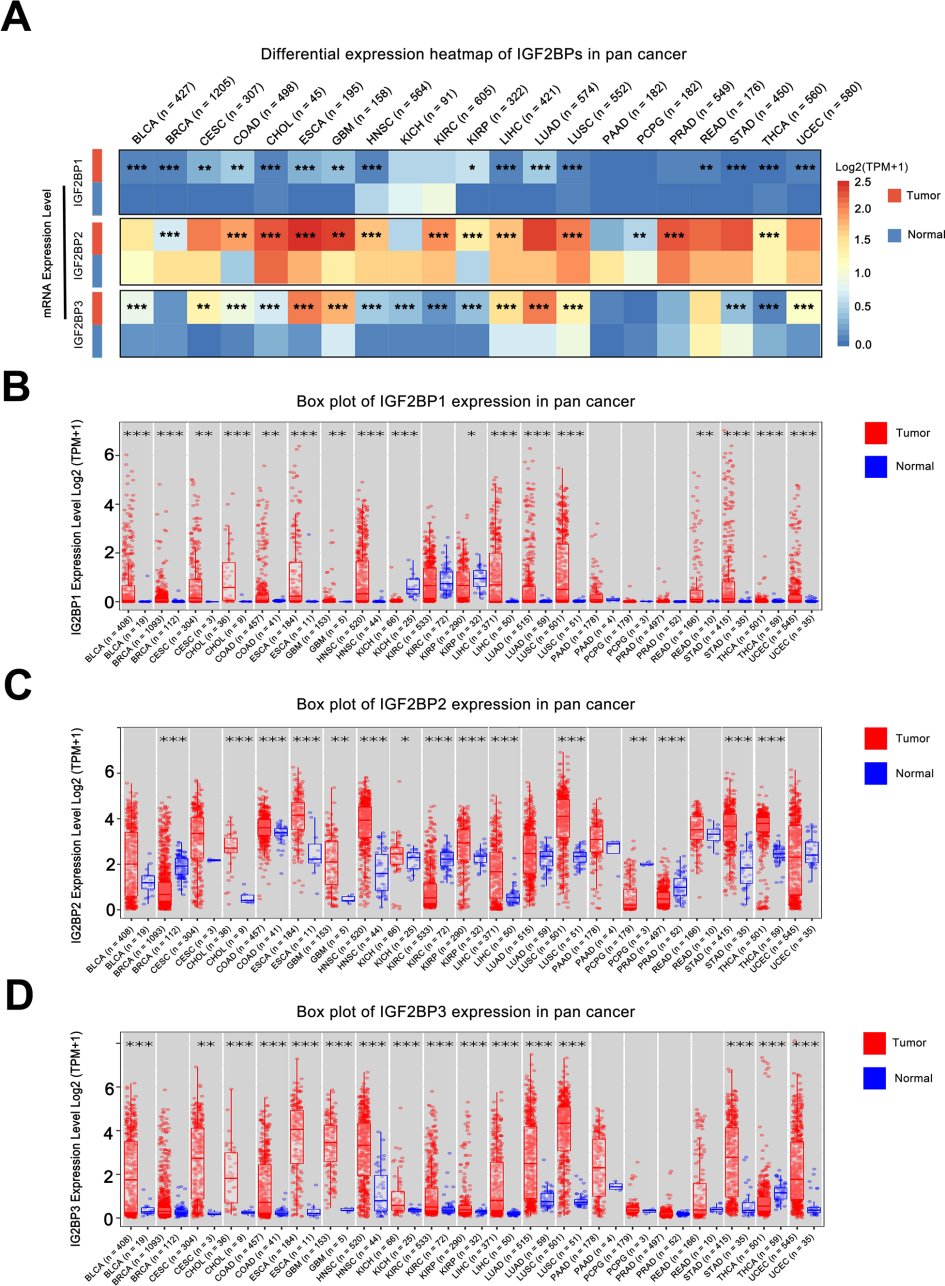
Differential expression of IGF2BPs gene family in different tumors. **A** Differential mRNA expression of IGF2BP1/2/3 between different tumor and adjacent normal tissues; **B**–**D** Box plots showing differences in IGF2BP1/2/3 mRNA expression between different tumor and adjacent normal tissues. (***P < 0.001, **P < 0.01, *P < 0.05)

The expression levels of IGF2BP1/2/3 varied between tumor tissues and adjacent normal tissues. In most tumors (CESC, COAD, ESCA, HNSC, LIHC, PRAD, etc.), the expression of IGF2BPs was significantly higher in tumor tissues compared to adjacent normal tissues (Fig. 2A). However, the expression patterns of IGF2BP1/2/3 were not completely consistent across different tumors. In tumors such as BRCA, PCPG, and PRAD, IGF2BP1 and IGF2BP3 expression was upregulated, while IGF2BP2 expression was downregulated. Furthermore, it was observed that IGF2BP3 expression was downregulated in THCA, while IGF2BP1 and IGF2BP2 were upregulated (Fig. 2B–D).

### Clinical significance of IGF2BPs gene family in multiple tumors

Analyzing the correlation among gene expression levels, patient survival time, and pathological stage can assist in predicting patient prognosis and assessing the influence of this gene on tumor progression.

**Fig. 3:**
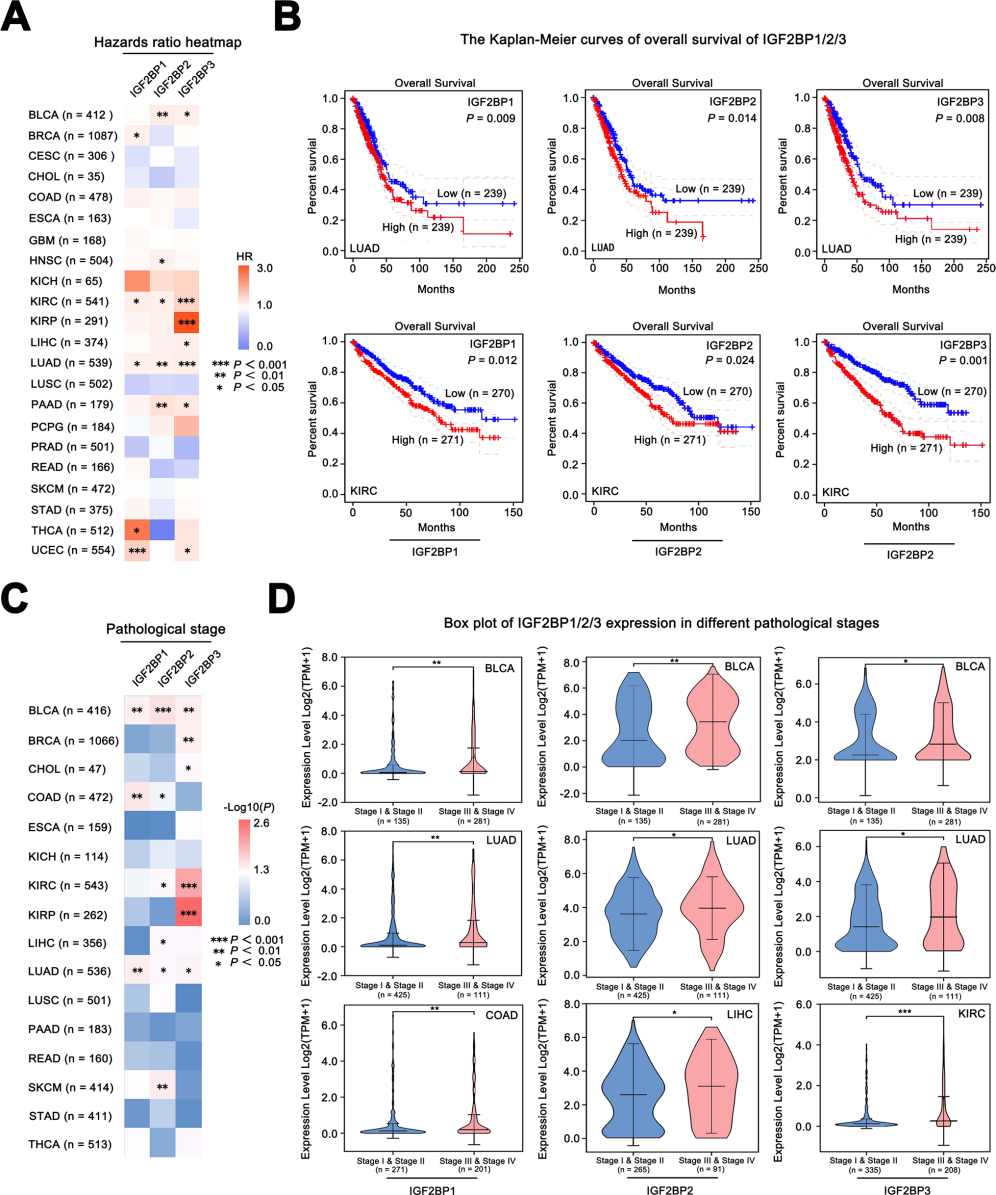
Clinical significance of IGF2BPs gene family in different tumors. **A** The HR heatmap displays the impact of IGF2BPs expression levels on overall survival in different tumors (with PRAD using PFS data). **B** The Kaplan–Meier curves of overall survival (OS) between low and high IGF2BP1/2/3 expression subgroups; **C** Significance heatmap indicating differences in IGF2BPs expression across different pathological stages; **D** Expression of IGF2BP1/2/3 in different pathological stages

Among nine tumors (BLCA, KIRC, KIRP, LIHC, LUAD, PAAD, PCPG, THCA, and UCEC), high expression of IGF2BP1/2/3 was identified as risk factors for poor prognosis in patients (Fig. 3A). For detailed information on the HR of various tumors, please refer to Supplementary Fig. 1. Upregulation of IGF2BP1/2/3 expression was associated with poor prognosis in both KIRC and LUAD (Fig. 3B). Additionally, in certain tumors (BLCA and LUAD), IGF2BP1/2/3 expression was correlated with the tumor pathological stage and increased with advancing stage (Fig. 3C-D). These results suggest that IGF2BPs may play a role in promoting tumor progression and support their potential as a diagnostic and prognostic markers.

### Genetic alterations of IGF2BPs gene family in different tumors

By analyzing the gene mutation status in different tumors, pathogenic genes that affect tumor development and growth can be identified, helping to reveal the pathogenesis of tumors.

**Fig. 4:**
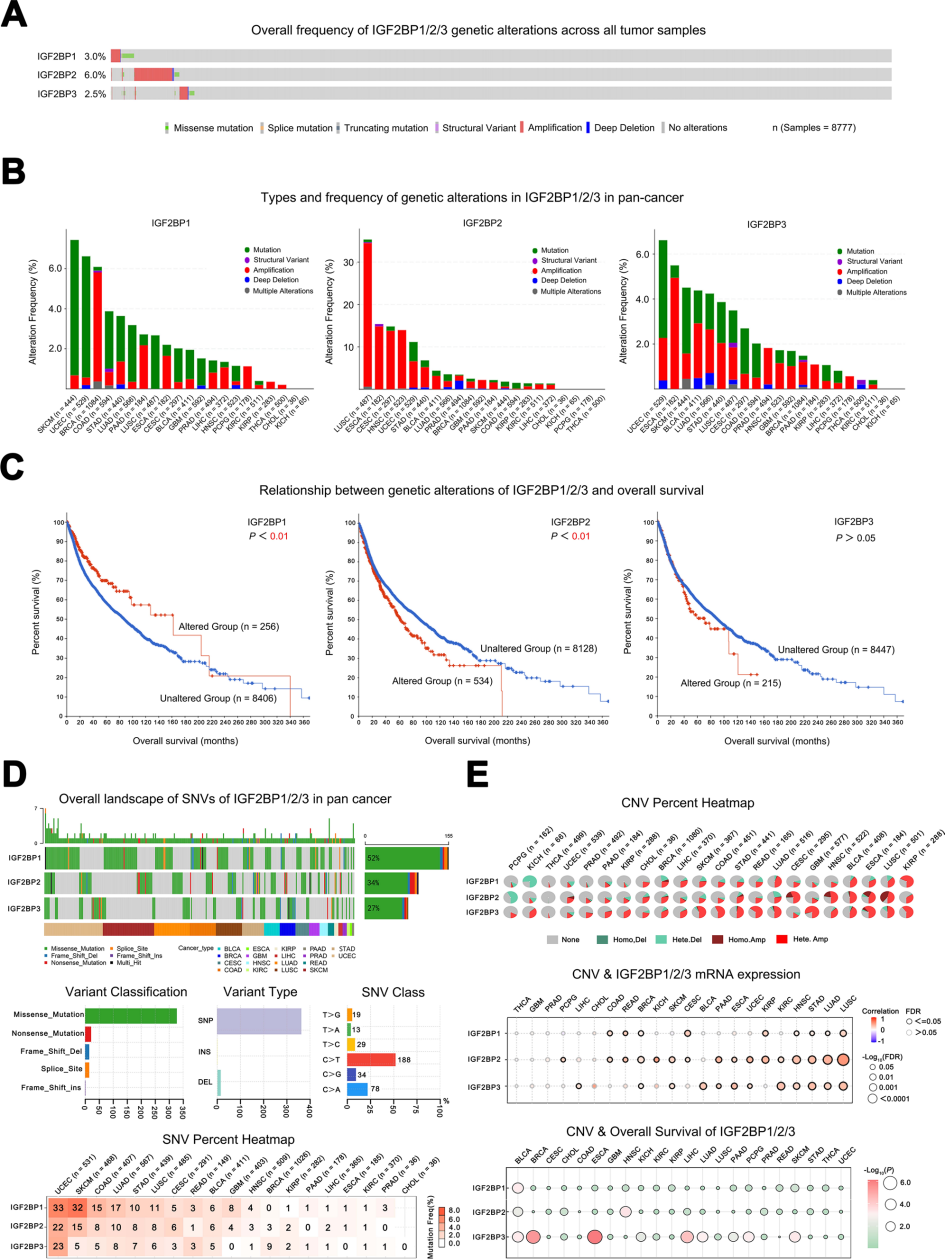
Genetic alterations of IGF2BPs gene family in different tumors. **A** Overall frequency of genetic alterations in IGF2BP1/2/3 in all tumor samples; **B** Types and frequency of genetic alterations in IGF2BP1/2/3 in diverse tumors; **C** Relationship between genetic alterations of IGF2BP1/2/3 and overall survival; **D** Overview of SNV in IGF2BP1/2/3 in various tumors; **E** Pie charts showing the distribution of heterozygous/homozygous CNV in IGF2BP1/2/3 in various tumors. The heatmap illustrates that the correlation between CNV and mRNA expression of IGF2BP1/2/3. The bubble plot depicts the impact of CNV on overall survival

The frequency of genetic alterations in IGF2BP1/2/3 was generally low (< 7%) in most tumors, with missense mutations and amplification mutations being the primary types (Fig. 4A). Notably, the frequency of gene amplification of IGF2BP2 exceeded 30% in LUSC (Fig. 4B). Additionally, mutations in IGF2BP2 are associated with the poor prognosis in patients, while mutations in IGF2BP1 appear to correlate with better outcomes. In contrast, mutations in IGF2BP3 did not significantly affect patient prognosis (Fig. 4C). The SNV status of IGF2BPs in different tumors revealed that the mutation from C to T was the primary variation type in their single nucleotide mutations (Fig. 4D).

The CNV of IGF2BPs (amplifications and deletions) was observed in most tumors. High-frequency homozygous amplifications were observed in LUSC, ESCA, HNSC, and CESC, while high-frequency heterozygous deletions occurred in KICH and PCPG. Furthermore, the CNV of IGF2BP1/2/3 showed a positive correlation with mRNA expression in most tumor types, while a negative correlation was observed in BRCA, CESC, COAD, KIRC, and LIHC. The CNV of IGF2BP1/2/3 was associated with the prognosis of various malignancies, such as UCEC, PRAD, and PAAD (Fig. 4E).

### Differential methylation levels of IGF2BPs gene family in several tumors

DNA methylation is a critical epigenetic modification that occurs widely in cells, regulating gene expression by attaching methyl groups to cytosine (C) precursor nucleotides on the DNA molecule [35]. In tumors, deviations in DNA methylation levels can lead to gene silencing or abnormal expression, contributing to the malignant evolution and proliferation of tumor cells [36]. Assessing the DNA methylation status of specific genes across different tumor types can provide insights into whether the methylation profiles of specific genes are associated with tumor classification, prognosis, and therapeutic reactions. Additionally, this analysis can illuminate the origins and mechanisms of tumor development.

**Fig. 5:**
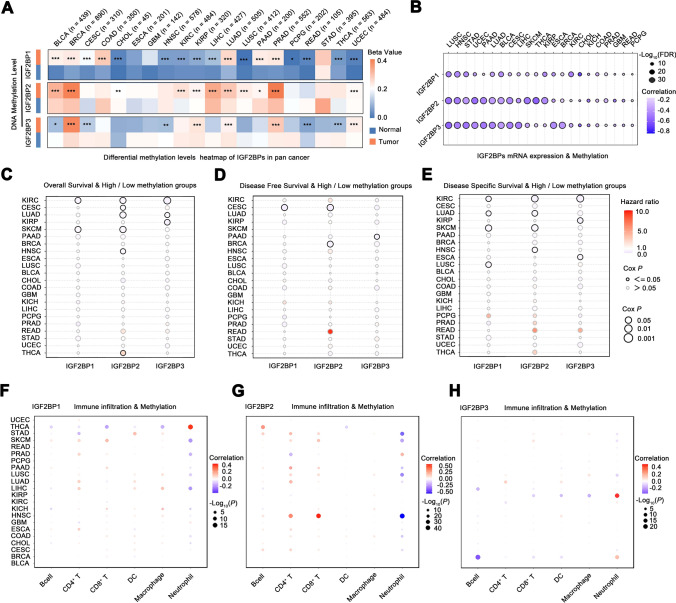
Differential methylation levels of IGF2BPs gene family in different tumors. **A** Methylation levels of IGF2BP1/2/3 between different tumor and adjacent normal tissues; **B** Correlation between IGF2BP1/2/3 expression and methylation level in different tumors; **C-E** The relationship between methylation levels of IGF2BP1/2/3 and clinical prognosis in various tumors; **F–H** Association between methylation levels of IGF2BP1/2/3 and immune cell infiltration in different tumors

The heatmap (Fig. 5A) illustrated significantly higher DNA methylation levels of IGF2BP1/2 in tumor tissues than in adjacent normal tissues in various tumors (BRCA, BLCA, COAD, LUAD, and PRAD). Conversely, the methylation levels of IGF2BP3 decreased in some tumors (BLCA, CESC, HNSC, READ, and THCA). Next, we analyzed the correlation between IGF2BP1/2/3 expression and methylation levels and found that IGF2BP1/2/3 expression was negatively correlated with methylation level in all tumors (Fig. 5B). Among these tumors, including KIRC, CESC, LUAD, KIRP, SKCM, PAAD, BRCA, HNSC, ESCA, and LUSC, individuals with low methylation levels of IGF2BP1/2/3 exhibited a higher risk of mortality. In THCA only, a high methylation level of IGF2BP2 was associated with an increased risk of mortality (Fig. 5C–E). In most tumors, the methylation levels of IGF2BP1/2 was positively correlated with B cell, CD4^+^ T, CD8^+^ T, DC, and Macrophages, while negatively correlated with neutrophils. In a variety of tumors, the methylation level of IGF2BP3 was negatively correlated with the infiltration of several immune cells (Fig. 5F–H).

### The GO and KEGG enrichment analysis on interacting protein-coding genes and co-expressed genes of IGF2BPs gene family

**Fig. 6:**
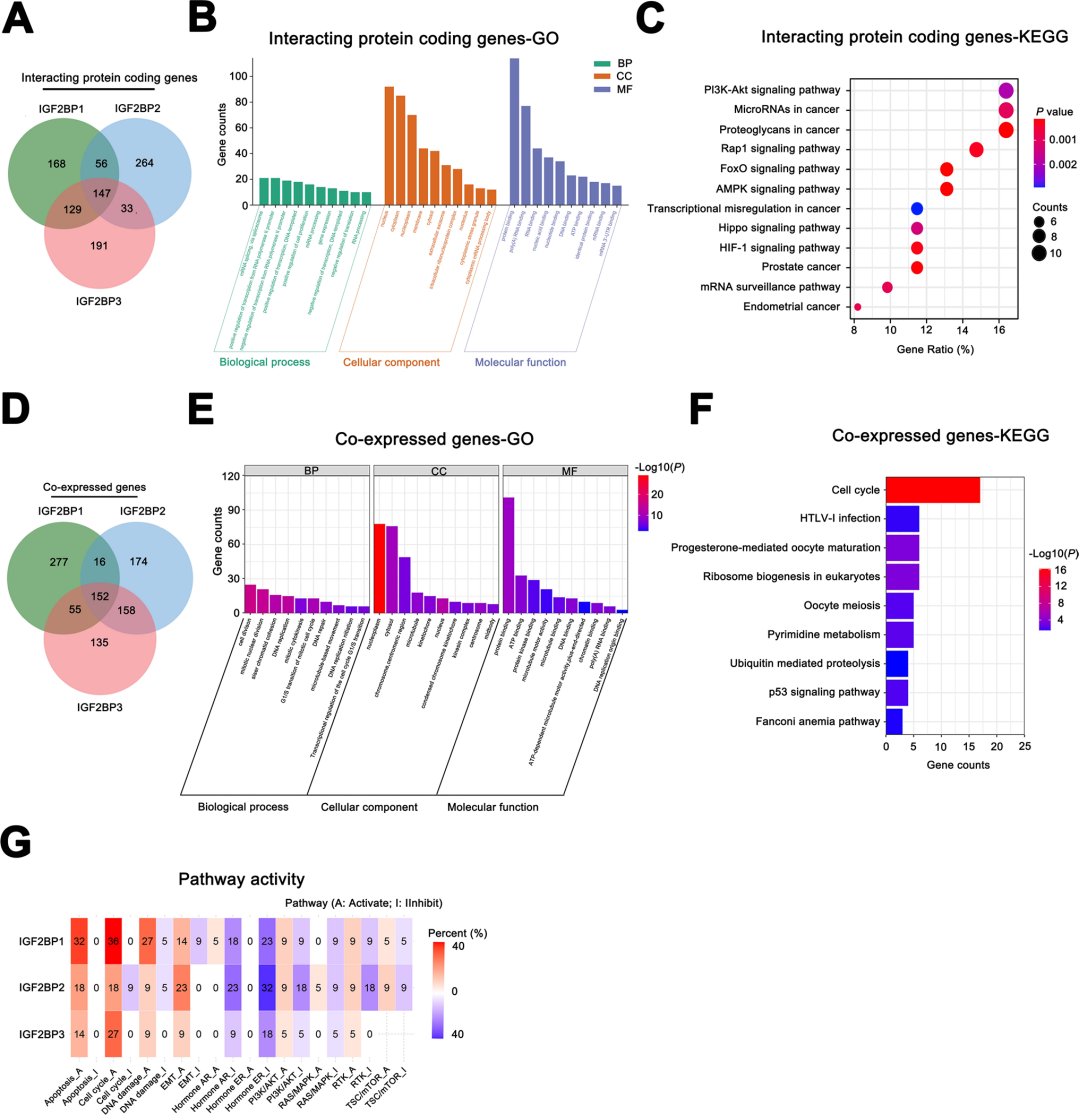
The GO and KEGG enrichment analysis on interacting protein-coding genes and co-expressed genes of IGF2BPs gene family. **A** The 147 common interacting protein-coding genes of IGF2BP1/2/3; **B**, **C** The GO and KEGG enrichment analysis of 147 common interacting protein-coding genes; **D** The 152 common co-expressed genes of IGF2BP1/2/3; **E**, **F** The GO and KEGG enrichment analysis of 152 co-expressed genes; **G** Correlation heatmap of IGF2BP1/2/3 expression and activation/inhibition status of 10 tumor-related pathways

Protein–protein interactions are fundamental to cellular signaling pathways. Analyzing the functions of proteins that interact with a specific gene can enhance our understanding of the biological processes and functions associated with that gene. We identified the intersection of the top 500 interacting protein-coding genes for IGF2BP1/2/3, resulting in 147 common interacting protein-coding genes (Fig. 6A). Subsequently, we performed the GO and KEGG enrichment analyses for the 147 genes. The GO enrichment analysis revealed that these genes were significantly enriched in biological processes related to transcription and translation (Fig. 6B). Meanwhile, the KEGG pathway analysis demonstrated their association with the activation of various tumor-related signaling pathways, including AMPK, Hippo, and PI3K-Akt (Fig. 6C).

Similarly, we focused on co-expressed genes of IGF2BP1/2/3. Co-expressed genes exhibit a strong consistency or correlation in expression levels, displaying a consistent pattern of synergistic changes. These genes are frequently involved in the same biological processes or pathways [37]. By conducting functional enrichment analysis, we can explore their roles and regulatory mechanisms in particular biological processes or signaling pathways. We performed GO and KEGG enrichment analyses on the 500 co-expressed genes for IGF2BP1/2/3. The results revealed their involvement in biological processes such as cell division, DNA replication and repair, cell cycle, and RNA transport (Supplementary Fig. 3A-B). We took the intersection of the top 500 co-expressed genes of IGF2BP1/2/3 and identified 152 common co-expressed genes (Fig. 6D). We then conducted GO and KEGG analyses on them. Our analyses found that these co-expressed genes were mainly involved in cell cycle regulation, cell proliferation and division, DNA and RNA replication, repair and metabolism, ubiquitin-mediated proteolysis and the p53 signaling pathway (Fig. 6E–F). Additionally, the pathway activity analysis indicated that IGF2BPs might play a role in regulating the cell cycle, DNA damage repair, and the activation of epithelial-mesenchymal transition (EMT) (Fig. 6G).

In summary, functional enrichment analysis of interacting protein-coding genes and co-expressed genes can reveal the biological processes involved in the genes of interest and their mechanisms in diseases.

### Association between IGF2BPs gene family expression and immune cell infiltration in different tumor microenvironments

In the tumor microenvironment, immune cells infiltrating tumors play a crucial role in tumor development, metastasis, and progression [38, 39]. The stromal score and immune score reflect the content of stromal and immune cell components in tumor tissues. Higher scores indicate a greater proportion of these components in the tumor microenvironment (TME). The estimated score reflects the proportion of tumor cell components in the TME and can be used to estimate tumor purity. Studying the relationship between gene expression levels and immune cell infiltration in tumors can illuminate the features of the TME, enhance our understanding of the role of genes in regulating immune cell infiltration and activity, and evaluate their potential as therapeutic targets for immunotherapy.

**Fig. 7:**
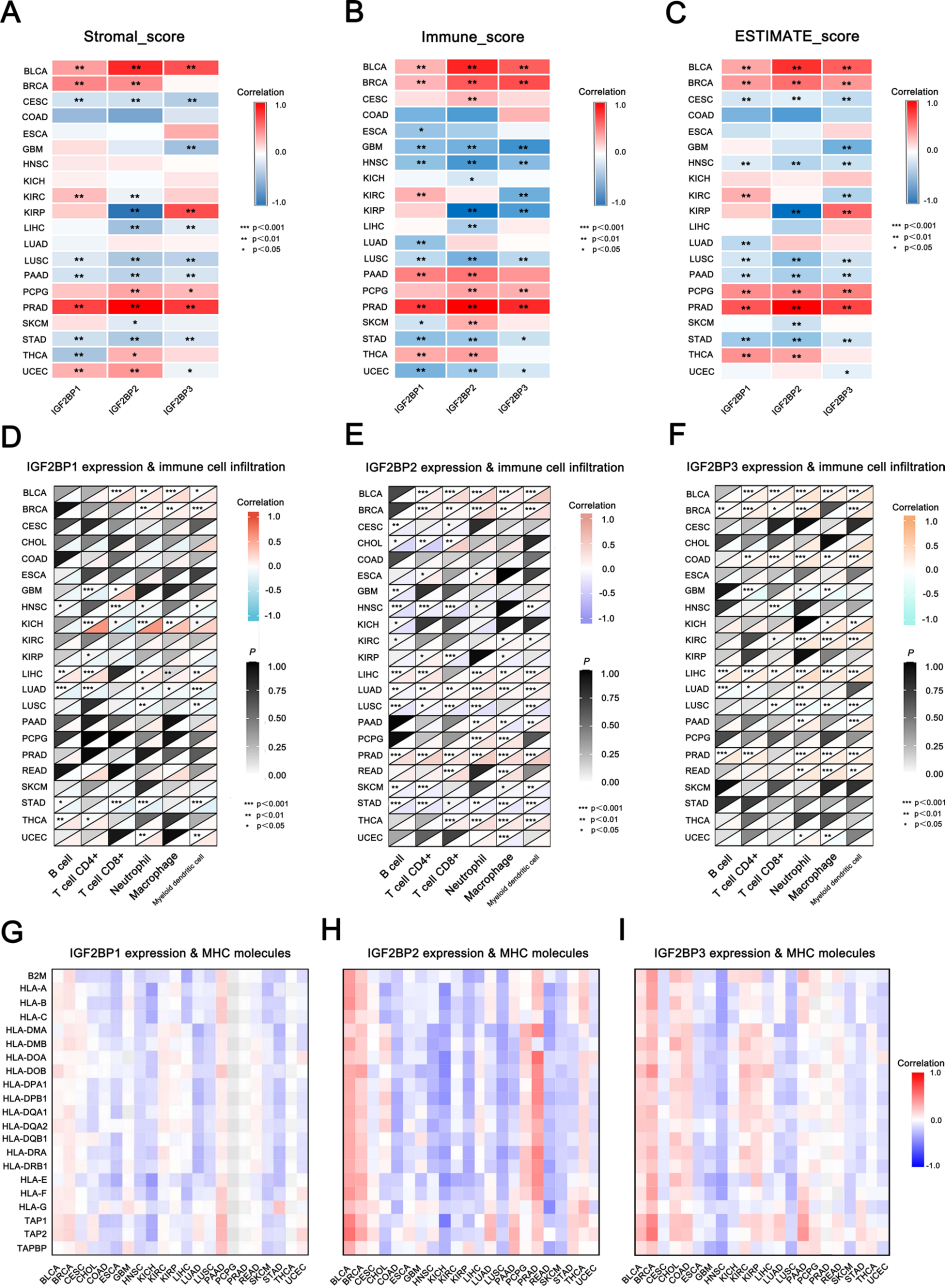
Association between IGF2BPs gene family expression and immune cell infiltration in different tumor microenvironments. **A**–**C** Stromal score, immune score, and estimate score of IGF2BP1/2/3 in different tumors; **D**–**F** Correlation between IGF2BP1/2/3 expression and immune cell infiltration; **G**–**I** Relationship between IGF2BP1/2/3 expression and MHC molecules

The correlation between IGF2BP1/2/3 expression and the stromal score was aligned with the immune score trend in several types of tumors (BRCA, BLCA, LUSC, PCPG, PRAD, STAD). However, an opposite trend was observed in CESC, PAAD, and UCEC. IGF2BP1/2/3 expression showed a positive correlation in BLCA, BRCA, PCPG, and PRAD, but a negative correlation in LUSC and STAD. Similarly, IGF2BP1/2/3 expression positively correlated with the estimated score in BLCA, BRCA, PCPG, PRAD, and THCA, while a negative correlation was found in CESC, HNSC, LUSC, PAAD, and STAD. Overall, the increase in IGF2BP1/2/3 expression might be accompanied by the infiltration of stromal cells and immune cells in the tumor immune microenvironment (Fig. 7A–C).

To better understand immune cell infiltration, we analyzed the correlation between IGF2BP1/2/3 expression and six types of immune cells (CD4 + T cell, CD8 + T cell, Neutrophil, Macrophage, and Myeloid dendritic cell). The correlation between IGF2BP1/2/3 expression and immune cell infiltration aligns with the immune score. In these tumors (BLCA, BRCA, and LIHC), IGF2BP1/2/3 expression was positively correlated with immune cell infiltration, while LUSC and STAD exhibited a negative correlation (Fig. 7D–F). These results indicated that immune infiltration varied among different types of tumors, and the expression of IGF2BP1/2/3 significantly correlated with immune cell levels across various tumors.

MHC is an important class of proteins on the cell surface that presents antigens to activate T cells, which initiates adaptive immune responses [40, 41]. MHC molecules are divided into two classes: MHC-I molecules present endogenous antigens to CD8 + T cells [42], facilitating the recognition and elimination of infected or abnormal cells, while MHC-II molecules present exogenous antigens to CD4 + T cells, stimulating humoral and helper immune responses [43]. MHC also plays a significant biological role in organ transplantation, autoimmune diseases, infections, and tumor immunity [44].

To further analyze the role of IGF2BPs in immune regulation and tumor immune microenvironment, we explored the association between IGF2BPs expression and MHC molecules in different tumors. We found that IGF2BP1/2/3 expression was positively correlated with MHC molecules in these tumors (BLCA, BRCA, and PAAD), while ESCA, HNSC, KICH, LUSC and SKCM exhibited a negative correlation (Fig. 7G–I). This phenomenon underscores the diversity of the tumor microenvironment and the various immune escape mechanisms present in different tumors types. This is highly significant for the immunological understanding of diseases and the development of treatment strategies.

### Assessing the role of IGF2BPs gene family in the efficacy of immunotherapy

Immune checkpoints are crucial in modulating the immune system's anti-tumor immunity through the activation of cytotoxic T cells (CTL) [45]. Immune checkpoint inhibitors, such as anti-CTLA-4 and anti-PD-1/anti-PD-L1, are frequently utilized in tumor immunotherapy [46]. Therefore, investigating the correlation between gene expression and immune checkpoint expression is essential for predicting the efficacy of immunotherapy, identifying novel therapeutic targets, and exploring the mechanisms of tumor immune evasion.

**Fig. 8:**
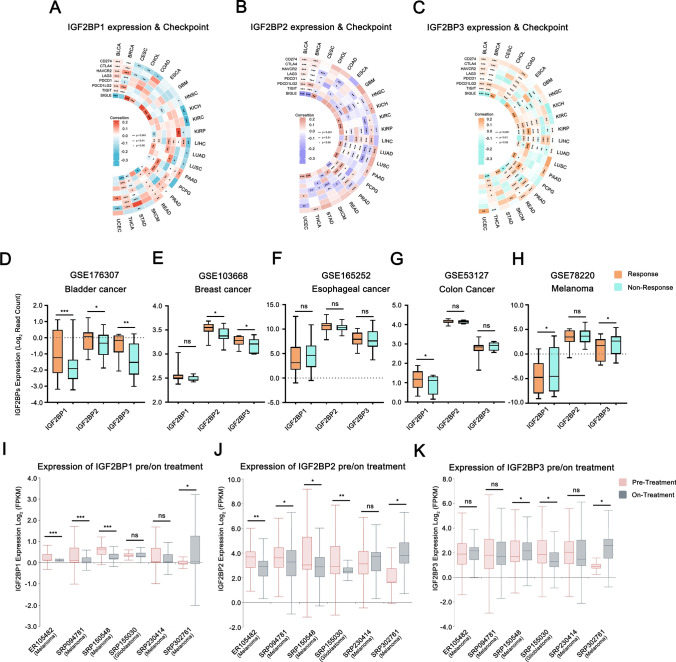
Assessing the role of IGF2BPs gene family in the efficacy of immunotherapy. **A**–**C** Correlation between immune checkpoints and IGF2BP1/2/3 expression; **D**–**H** Expression of IGF2BP1/2/3 in immunotherapy responders and non-responders; **I**–**K** Expression of IGF2BP1/2/3 in patients before and after immunotherapy

The expression of IGF2BP1/2/3 was closely correlated with immune checkpoint activity. Certain tumors (BLCA, BRCA, LIHC, and PRAD) displayed a positive correlation, while others (CESC, HNSC, LUAD, and LUSC) showed a negative correlation (Fig. 8A–C). High expression of IGF2BP1/2/3 was associated with increased levels of immunosuppression-related markers (PD-L1 and CTLA-4) in tumors. We also analyzed multiple GEO datasets to explore the relationship between the expression of the IGF2BP1/2/3 and response to immunotherapy. In bladder cancer, breast cancer, and colon cancer, higher IGF2BP1/2/3 expression was observed in immunotherapy responders compared to non-responders, while the opposite trend was observed in melanoma. In esophageal cancer, there was no significant difference in the expression of IGF2BP1/2/3 between immune responders and non-responders. Previous results also indicated that the expression of IGF2BP1/2/3 in ESCA was not significantly correlated with immune checkpoint activity (Fig. 8D–H). In addition, we analyzed the expression changes of IGF2BP1/2/3 in melanoma and glioblastoma patients before and after anti-PDL1 immunotherapy through multiple SRA datasets. The results showed that after immunotherapy, the expression of IGF2BP1/2/3 was significantly downregulated in melanoma and glioblastoma patients, except in the SRP302761 dataset (melanoma) (Figs. 8I–K).

### IGF2BPs gene family expression correlates with TMB/MSI and drug sensitivity

**Fig.9:**
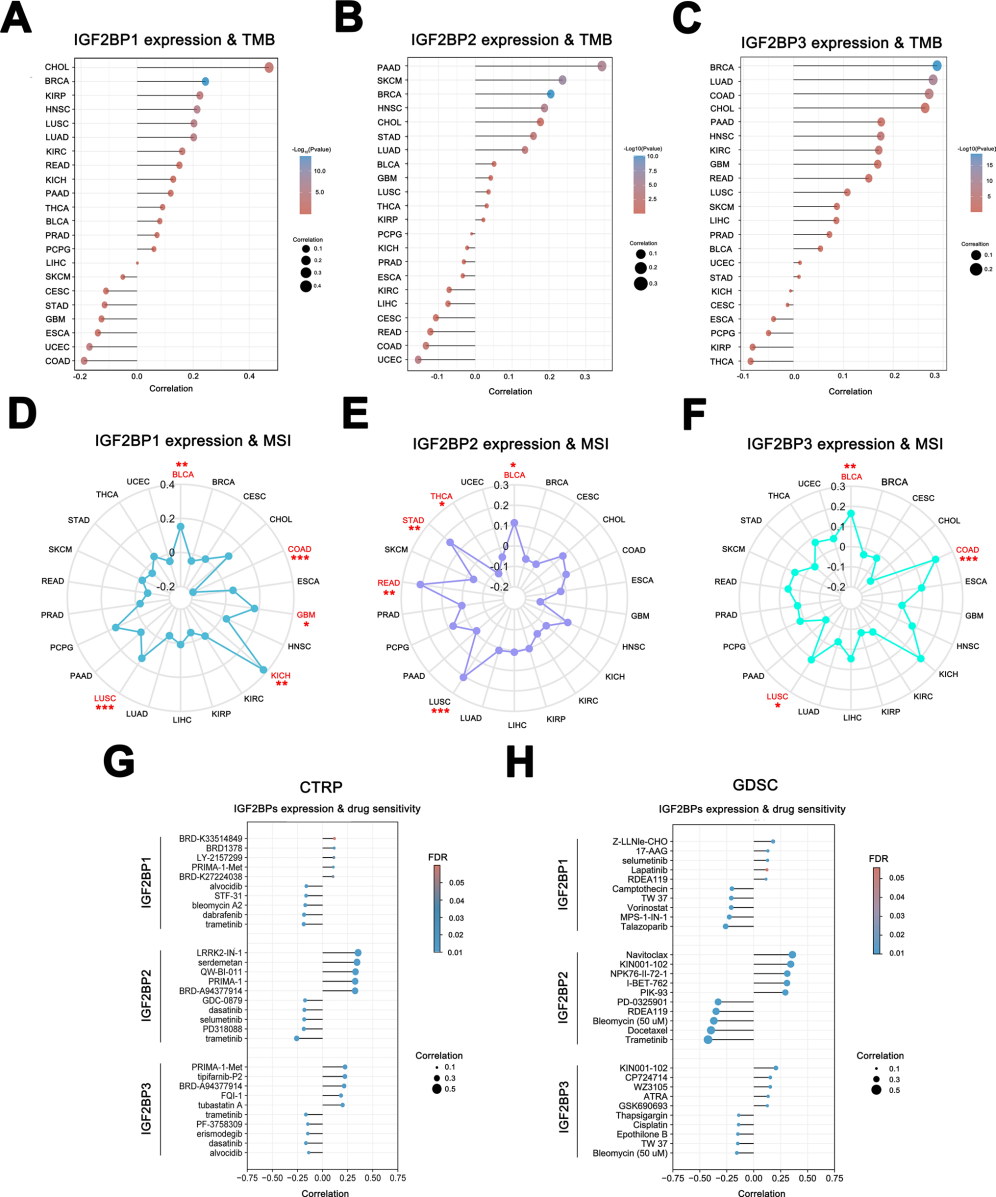
IGF2BPs gene family expression correlates with TMB/MSI and drug sensitivity. **A**–**C** Correlation between IGF2BP1/2/3 expression and TMB; **D**–**F** Relationship between IGF2BP1/2/3 expression and MSI; **G**–**H** Relationship between IGF2BP1/2/3 expression and drug sensitivity

Immunotherapy has become an effective cancer treatment approach, using key indicators such as Tumor Mutational Burden (TMB) and Microsatellite Instability (MSI) commonly to predict the efficacy of immunotherapy. TMB refers to the extent and quantity of mutations present in tumor cells, while MSI indicates dysregulation of microsatellite DNA sequence repeats in tumor cells [47, 48]. TMB and MSI serve as biomarkers for predicting the efficacy of immunotherapy, with tumors exhibiting high TMB and MSI may respond better to immunotherapy. In some tumors (such as BLCA, LUSC, LUAD, HNSC, CHOL, and PAAD), the expression of IGF2BP1/2/3 is significantly positively correlated with TMB, but negatively correlated in CESC and ESCA (Fig. 9A-C). Additionally, certain tumors (BRCA, COAD, LUSC) showed a significant correlation between IGF2BP1/2/3 expression and MSI (Fig. 9D-F). These results suggested that the effectiveness of IGF2BP as an immunotherapy target may vary in different types of tumors.

Analyzing the correlation between drug and gene expression can help explore the mechanisms of drug resistance or sensitivity in tumors, thereby enhancing the efficacy of related drug treatments. As shown in figures, the top 5 sensitive/resistant drugs significantly associated with IGF2BP1/2/3 were identified from the CTRP and GDSC databases. Data from the CTRP database indicated a negative correlation between the expression of IGF2BP1/2/3 and the IC50 values of alvocidib, dasatinib, and trametinib, suggesting higher drug sensitivity. Conversely, the IC50 value of BRD-A94377914 (an HDAC inhibitor) showed a positive correlation, indicating drug resistance. The data from the GDSC database aligned with the CTRP data, displaying the sensitivity of IGF2BP1/2/3 to trametinib (Fig. 9G-H), which is commonly used for treating specific cancer types like LUSC and SKCM. These findings suggested that the IGF2BPs gene family could serve as effective targets for drug therapy in certain tumors.

## Discussion

Although numerous studies have established the tumor-promoting functions of the IGF2BPs gene family, their exact roles in various tumor types remain controversial. Consequently, we conducted a comprehensive pan-cancer analysis to explore their role across different tumor types. This analysis aims to enhance our understanding of the expression patterns and functional characteristics of these proteins in tumors, thereby offering valuable insights for tumor diagnosis, treatment approaches, and prognosis assessment.

Differential expression analysis showed abnormally high expression of IGF2BPs in most tumors. Specifically, IGF2BP1 and IGF2BP3 exhibited lower expression levels in different tumors and adjacent normal tissues compared to IGF2BP2. Previous studies have also reported that IGF2BP family proteins promote malignant phenotypes including tumor cell proliferation, invasion, and metastasis [49–51]. Our survival analysis results revealed that high expression of IGF2BPs was associated with poor prognosis in multiple tumors, such as BLCA, KIRC, LUAD, PAAD, and UCEC.

The amplification or mutation of genes is widely recognized as increasing susceptibility to tumors and facilitating tumor initiation and progression [52]. There is evidence that overexpression of IGF2BPs caused by mutations can promote tumor progression. In thyroid cancer, the fusion of THADA gene with LOC389473 gene leads to abnormal IGF2BPs expression, activating the IGF1R signaling pathway, and promoting the proliferation and invasion of cancer cells [53]. However, our mutation analysis results indicated that the mutation frequency of IGF2BPs was low in most tumors, and its correlation with patient prognosis was not significant. Interestingly, amplification mutations of IGF2BP2 were found in more than 30% of LUSC, but these were not significantly associated with patient prognosis (Supplementary Fig. 2A–C). Furthermore, our analysis revealed no significant correlation between the expression of IGF2BP2 and various clinical parameters including age, gender, race, TNM stage, pathological stage, prognosis (OS, DSS and PFS) and treatment outcomes (Supplementary Table 7), which is consistent with findings from prior studies [54].

Our methylation analysis results showed the methylation levels of IGF2BP1/2 were elevated compared to adjacent normal tissues, while the methylation levels of IGF2BP3 were decreased in most tumors. Nevertheless, the overall levels of methylation for IGF2BP1/2/3 tended to be moderate to low rather than high in tumors. Interestingly, our study found negative correlations between the expression levels of IGF2BP1/2/3 and methylation levels in all tumors. It is well known that under normal circumstances, high methylation levels in a gene region reduce the accessibility of the region, causing the expression of the gene to be inhibited, resulting in a reduction in gene expression levels [55]. DNA methylation can prevent the binding of transcription factors to gene promoters, promote chromatin compaction, and hinder the binding of polymerases, thereby reducing the transcriptional activity of genes and ultimately affecting gene expression [56]. In addition, histone modifications can enhance the accessibility of the IGF2BPs gene promoters by altering chromatin structure, thereby increasing their expression. Certain histone methylation, such as H3K27me3, may inhibit the expression of IGF2BPs [57]. Furthermore, histone modifications can regulate the binding affinity of transcription factors, thus affecting their regulation and expression of the IGF2BPs promoters [58]. Our results found that IGF2BP1/2/3 was abnormally highly expressed in most tumors, but the DNA methylation levels were not high, consistent with previous studies. Low methylation levels of IGF2BP1/2/3 have been associated with poor prognosis in some tumors (KIRC, CESC, LUAD, SKCM, KIRP and HNSC), potentially due to the activation and expression of IGF2BPs. This activation may enhance the transcription tumors-related genes, thereby promoting cancer development. However, in THCA, high levels of IGF2BP2 hypermethylation indicated a poor prognosis. This could be due to elevated levels of methylation suppressing the expression of tumor suppressor genes, resulting in uncontrolled tumor cell proliferation and spread, ultimately impacting the prognosis of patients.

Given the high homology among the members of the IGF2BPs family, we conducted functional enrichment analysis on their co-expressed genes and genes encoding interacting proteins to elucidate the biological processes and potential mechanisms involved in the collective actions of IGF2BPs. In our functional enrichment analysis, we identified several common interacting protein-coding genes within the IGF2BPs gene family including IGF2, HMGA2, and LIN28A/B, all of which are known to facilitate tumor progression [59, 60]. Our enrichment analysis indicated that IGF2BP1/2/3 were involved in transcription and translation regulation, modulation cell cycle, and facilitatin cell division and proliferation, DNA damage repair, and activation of epithelial-mesenchymal transition. Additionally, IGF2BPs can impact various common downstream signaling pathways such as AMPK, Hippo, PI3K-Akt, RaP1, and p53.

Immune correlation analysis revealed that high expression of IGF2BPs was positively correlated with immune cell infiltration in various tumors. Additionally, IGF2BPs expression can influence the efficacy of immune checkpoint inhibitors. Interestingly, our study found that high expression of IGF2BPs in BLCA, BRCA, and PAAD may be linked to the upregulation of MHC molecules. This suggested that IGF2BPs could enhance antigen presentation capabilities, activate T cells, and increase the infiltration of immune cells, which is consistent with the previous immune cell infiltration results. This contributes to anti-tumor immune responses, facilitating the recognition and elimination of tumor cells, and inhibiting tumor growth and metastasis in these tumors. On the other hand, in certain other tumors (ESCA, HNSC, KICH, LUSC and SKCM), elevated IGF2BPs may suppress MHC molecules expression, resulting in reduced antigen presentation ability and decreased infiltration of immune cells, including T cells and natural killer cells (NK cells). This indicated that IGF2BPs may promote tumor growth and metastasis by evading immune surveillance through the suppression of immune responses in these tumors. Moreover, this indicated that IGF2BPs expression may have varying biological effects across different tumor types, highlighting their complex interplay with the tumor immune microenvironment. This complexity underscored the need for further investigation into the role of IGF2BPs in pan-cancer contexts. Furthermore, our results were consistent with a previous study on melanoma [61], where patients treated with PD-1 antibody immunotherapy exhibited significantly decreased IGF2BP1 expression after treatment. Conversely, in a study on breast cancer, IGF2BP3 expression levels were significantly upregulated in patients who responded well to immunotherapy [62]. Furthermore, the expression of IGF2BPs was closely related to immunotherapy sensitivity indicators (TMB/MSI). Several studies have demonstrated a strong correlation between high levels of TMB/MSI and the mutational processes of tumors and the pathways involved in DNA repair [63], which was further validated by our functional enrichment analysis results. This information indicates that the efficacy of immunotherapy with IGF2BPs may vary depending on the tumor type. Furthermore, alterations in the expression of IGF2BP1/2/3 can potentially activate or inhibit the immune checkpoints, thereby influencing anti-tumor response. IGF2BPs can regulate the immune microenvironment, playing a vital role in tumor immunotherapy, and may serve as potential targets for immunotherapy. However, translating IGF2BPs into cliniccal targets for immunotherapy requires further exploration. First, developing IGF2BPs as biomarkers could assist in identifying patient populations best suited for immunotherapy, thus enhancing treatment precision. Second, we should investigate small molecule drugs or antibodies that target IGF2BPs in conjunction with existing immunotherapy strategies, such as PD-1/PD-L1 inhibitors, to boost immune responses. Additionally, it's essential to design rigorous clinical trials to assess the safety and efficacy of IGF2BPs-targeted therapies, allowing us to collect the clinical data necessary to support their application. Despite the complexities of individualized treatment and challenges in clinical validation during the transformation process, in-depth research on IGF2BP not only provides important clues for us to understand cancer immune mechanisms, but also offers new approaches for the development of future cancer treatments.

Analysis of drug sensitivity revealed a negative correlation between the expression of IGF2BPs and several common chemotherapy drugs (alvocidib, dasatinib, trametinib, and selumetinib), indicating that elevated expression levels of IGF2BPs have a detrimental impact on the efficacy of these drugs. A study has demonstrated that alterations in IGF2BP2 expression are linked to dasatinib resistance in non-small cell lung cancer cells [64]. Moreover, another study indicated that the expression of IGF2BP1 impacts the responsiveness of melanoma cells to dabrafenib and trametinib [65]. Some of these drugs are MEK signaling pathway inhibitors, which can interfere with cell proliferation and growth to treat tumors. Previous studies have also confirmed that IGF2BPs can aberrantly activate the MEK pathway, promoting cancer cell proliferation and invasion, thus mediating tumor progression [66]. Therefore, by using MEK-inhibiting drugs on tumors, the activation of IGF2BPs in the MEK pathway can be prevented, resulting in effective drug treatment. These results suggest that the IGF2BPs may serve as effective targets for tumor drug therapy.

Based on this study and existing literature, we analyzed the roles of the IGF2BPs gene family in various tumor types and found that they exhibit contradictory functions, which can be attributed to several factors. First, the functional diversity among IGF2BPs gene family leads to their distinct roles in different cancers. For instance, IGF2BP1 typically promotes tumor growth in breast [67] and liver cancers [68], while IGF2BP2 and IGF2BP3 are involved in enhancing the proliferation and metastasis of certain cancers, such as colon cancer [69]. Second, the expression and activity of IGF2BPs can be influenced by the specific microenvironments of various tumors. Each tumor type possesses unique signaling pathways, tumor-promoting factors, and immune microenvironments, which result in differential effects of IGF2BPs. For example, in the microenvironment of gastric cancer, cancer-associated fibroblasts modulate the immune response of tumor cells and promote a malignant phenotype by regulating IGF2BP3 expression [70]. Additionally, the expression of IGF2BPs is subject to complex regulatory mechanisms, such as epigenetic and transcriptional regulation. For example, IGF2BP2 expression in colorectal cancer is influenced by DNA methylation [71]. Furthermore, the interactions between IGF2BP1, IGF2BP2, and IGF2BP3 with various RNAs and proteins dictate their roles across different tumors. For example, IGF2BP2 promotes tumor development in liver cancer by binding to mRNAs with stem cell characteristics, such as OCT4 [72]. Finally, clinical studies reveal that the relationship between IGF2BPs and patient prognosis varies significantly across different cancers. High expression levels may correlate with poor prognosis in some malignancies, whereas in others, the opposite is true. In summary, these factors commonly contribute to the contradictory biological effects of the IGF2BPs gene family across various tumors. The intricate interplay allows the IGF2BPs gene family to assume diverse and dynamic roles in tumor biology, and further research will enhance our understanding of its specific mechanisms in cancer.

Although we have obtained the differences in mRNA expression and DNA methylation levels of the IGF2BPs gene family in different tumors, as well as the mutation status and close association with poor prognosis in patients, pathological stage, and immune-related indicators (such as immune cell infiltration, immune checkpoints, TMB, MSI and MHC). Additionally, it was found that they may promote tumor progression through common signaling pathways. However, our study still has some limitations. First, there is an inevitable introduction of systematic bias from using different public datasets. Such bias can result in the overestimation or underestimation of findings for certain cancer types, potentially compromising the objectivity of the conclusions. Therefore, it is essential to employ statistical methods for correction and data normalization when integrating information from various sources to ensure the reliability and consistency of the results. Second, the biological heterogeneity existing among different tumors types, subtypes, and individuals may influence the conclusions drawn from our analysis and their applicability. This heterogeneity may result in research conclusions being applicable to certain cancer types but not to others. In future studies, it is essential to employ a stratified analysis strategy to conduct more detailed comparisons across different cancer types and subtypes. Such an approach will yield more targeted and universally applicable results, and provide a comprehensive understanding of the biological functions of the IGF2BPs gene family in various cancer. Furthermore, using relevant in vitro and in vivo models is necessary to validate the biological functions of the IGF2BPs gene family in different tumors and their potential mechanisms. Without experimental validation, conclusions may rely too heavily on existing data, leading to misconceptions about their biological functions. Additionally, the relationship between the IGF2BPs gene family and clinical parameters (such as patient prognosis and pathological stage) in different tumors needs to be verified through a large number of clinical samples. The characteristics of different patient populations can influence the expression of IGF2BPs and their relationship with tumor progression. Therefore, relying solely on public datasets may not fully capture their clinical significance. Finally, immune-related experiments are required to demonstrate the potential of the IGF2BPs gene family as targets for immunotherapy. The absence of relevant immunological experiments may result in an incomplete assessment of the research's clinical applicability, thus impacting its potential value in clinical practice.

## Conclusion

In conclusion, our results support previous conclusions that the IGF2BPs gene family acts as an oncogene to promote tumor progression and significantly impacts patient prognosis. Our research findings demonstrate that IGF2BPs regulate post-transcriptional modifications by recognizing and binding to m6A-modified mRNA, participating in biological processes such as cell cycle changes, cell proliferation and division, DNA/RNA replication, transcription, translation and metabolism, DNA damage repair, and the activation of EMT and related pro-tumor signaling pathways such as AMPK, Hippo, PI3K-Akt, and p53. Therefore, the abnormally high expression of IGF2BPs in tumors regulates immune microenvironments and immune checkpoints, inhibits immune infiltration, enhances malignant phenotypes, including cancer cell proliferation, cell cycle alterations, invasion and metastasis, drug resistance, and immune escape, thereby promoting tumor progression.

The high expression of IGF2BPs in tumors has a detrimental impact on patient prognosis and plays a significant role in regulating the immune microenvironment and immune checkpoints. Therefore, the IGF2BPs gene family has the potential as a biomarker for early cancer diagnosis and prognosis and as an immunotherapy target. In summary, conducting pan-cancer analyses of IGF2BPs can help understand their mechanism across different tumors, providing new insights and strategies for tumor prevention and treatment.

## Supplementary Information

Additional file 1.

## Data Availability

The databases referenced in the methods section of this article are all open access. Data is provided within the manuscript or supplementary information files.

## References

[1] Tian S, Lai J, Yu T, Li Q, Chen Q. Regulation of gene expression associated with the N6-methyladenosine (m6A) enzyme system and its significance in cancer. Front Oncol. 2020;10: 623634. 33552994 10.3389/fonc.2020.623634 PMC7859513

[2] Fang Z, Mei W, Qu C, Lu J, Shang L, Cao F, Li F. Role of m6A writers, erasers and readers in cancer. Exp Hematol Oncol. 2022;11:45. 35945641 10.1186/s40164-022-00298-7 PMC9361621

[3] He L, Li H, Wu A, Peng Y, Shu G, Yin G. Functions of N6-methyladenosine and its role in cancer. Mol Cancer. 2019;18:176. 31801551 10.1186/s12943-019-1109-9 PMC6892141

[4] Duan M, Liu H, Xu S, Yang Z, Zhang F, Wang G, Wang Y, Zhao S, Jiang X. IGF2BPs as novel m(6)A readers: diverse roles in regulating cancer cell biological functions, hypoxia adaptation, metabolism, and immunosuppressive tumor microenvironment. Genes Dis. 2024;11:890–920. 37692485 10.1016/j.gendis.2023.06.017 PMC10491980

[5] Huang H, Weng H, Sun W, Qin X, Shi H, Wu H, Zhao BS, Mesquita A, Liu C, Yuan CL, Hu YC, Hüttelmaier S, Skibbe JR, et al. Recognition of RNA N(6)-methyladenosine by IGF2BP proteins enhances mRNA stability and translation. Nat Cell Biol. 2018;20:285–95. 29476152 10.1038/s41556-018-0045-z PMC5826585

[6] Nielsen FC, Nielsen J, Christiansen J. A family of IGF-II mRNA binding proteins (IMP) involved in RNA trafficking. Scand J Clin Lab Invest Suppl. 2001;234:93–9. 11713986

[7] Bell JL, Wächter K, Mühleck B, Pazaitis N, Köhn M, Lederer M, Hüttelmaier S. Insulin-like growth factor 2 mRNA-binding proteins (IGF2BPs): post-transcriptional drivers of cancer progression? Cell Mol Life Sci. 2013;70:2657–75. 23069990 10.1007/s00018-012-1186-z PMC3708292

[8] Nielsen J, Kristensen MA, Willemoës M, Nielsen FC, Christiansen J. Sequential dimerization of human zipcode-binding protein IMP1 on RNA: a cooperative mechanism providing RNP stability. Nucleic Acids Res. 2004;32:4368–76. 15314207 10.1093/nar/gkh754 PMC514376

[9] Nielsen J, Christiansen J, Lykke-Andersen J, Johnsen AH, Wewer UM, Nielsen FC. A family of insulin-like growth factor II mRNA-binding proteins represses translation in late development. Mol Cell Biol. 1999;19:1262–70. 9891060 10.1128/mcb.19.2.1262 PMC116055

[10] Wächter K, Köhn M, Stöhr N, Hüttelmaier S. Subcellular localization and RNP formation of IGF2BPs (IGF2 mRNA-binding proteins) is modulated by distinct RNA-binding domains. Biol Chem. 2013;394:1077–90. 23640942 10.1515/hsz-2013-0111

[11] Jia M, Gut H, Chao JA. Structural basis of IMP3 RRM12 recognition of RNA. RNA. 2018;24:1659–66. 30135093 10.1261/rna.065649.118 PMC6239170

[12] Lunde BM, Moore C, Varani G. RNA-binding proteins: modular design for efficient function. Nat Rev Mol Cell Biol. 2007;8:479–90. 17473849 10.1038/nrm2178 PMC5507177

[13] Cao J, Mu Q, Huang H. The roles of insulin-like growth factor 2 mRNA-binding protein 2 in cancer and cancer stem cells. Stem Cells Int. 2018;2018:4217259. 29736175 10.1155/2018/4217259 PMC5874980

[14] Huang X, Zhang H, Guo X, Zhu Z, Cai H, Kong X. Insulin-like growth factor 2 mRNA-binding protein 1 (IGF2BP1) in cancer. J Hematol Oncol. 2018;11:88. 29954406 10.1186/s13045-018-0628-y PMC6025799

[15] Zhou H, Sun Q, Feng M, Gao Z, Jia S, Cao L, Yu X, Gao S, Wu H, Li K. Regulatory mechanisms and therapeutic implications of insulin-like growth factor 2 mRNA-binding proteins, the emerging crucial m(6)A regulators of tumors. Theranostics. 2023;13:4247–65. 37554271 10.7150/thno.86528 PMC10405845

[16] Shao W, Zhao H, Zhang S, Ding Q, Guo Y, Hou K, Kan Y, Deng F, Xu Q. A pan-cancer landscape of IGF2BPs and their association with prognosis, stemness and tumor immune microenvironment. Front Oncol. 2022;12:1049183. 36686749 10.3389/fonc.2022.1049183 PMC9846525

[17] Azzam SK, Alsafar H, Sajini AA. FTO m6A demethylase in obesity and cancer: implications and underlying molecular mechanisms. Int J Mol Sci. 2022;23:3800. 35409166 10.3390/ijms23073800 PMC8998816

[18] Jiang X, Liu B, Nie Z, Duan L, Xiong Q, Jin Z, Yang C, Chen Y. The role of m6A modification in the biological functions and diseases. Signal Transduct Target Ther. 2021;6:74. 33611339 10.1038/s41392-020-00450-x PMC7897327

[19] Huang H, Weng H, Chen J. m(6)A modification in coding and non-coding RNAs: roles and therapeutic implications in cancer. Cancer Cell. 2020;37:270–88. 32183948 10.1016/j.ccell.2020.02.004 PMC7141420

[20] Li Y, Gu J, Xu F, Zhu Q, Chen Y, Ge D, Lu C. Molecular characterization, biological function, tumor microenvironment association and clinical significance of m6A regulators in lung adenocarcinoma. Brief Bioinform. 2021;22:bbaa225. 33003204 10.1093/bib/bbaa225

[21] Waly AA, El-Ekiaby N, Assal RA, Abdelrahman MM, Hosny KA, El Tayebi HM, Esmat G, Breuhahn K, Abdelaziz AI. Methylation in MIRLET7A3 gene induces the expression of IGF-II and its mRNA binding proteins IGF2BP-2 and 3 in hepatocellular carcinoma. Front Physiol. 2018;9:1918. 30733684 10.3389/fphys.2018.01918 PMC6353855

[22] Zhang L, Wan Y, Zhang Z, Jiang Y, Gu Z, Ma X, Nie S, Yang J, Lang J, Cheng W, Zhu L. IGF2BP1 overexpression stabilizes PEG10 mRNA in an m6A-dependent manner and promotes endometrial cancer progression. Theranostics. 2021;11:1100–14. 33391523 10.7150/thno.49345 PMC7738899

[23] Tang H, Zhao J, Liu J. Comprehensive analysis of the expression of the IGF2BPs gene family in head and neck squamous cell carcinoma: association with prognostic value and tumor immunity. Heliyon. 2023;9: e20659. 37842569 10.1016/j.heliyon.2023.e20659 PMC10568114

[24] Sun T, Wu R, Ming L. The role of m6A RNA methylation in cancer. Biomed Pharmacother. 2019;112: 108613. 30784918 10.1016/j.biopha.2019.108613

[25] Du QY, Zhu ZM, Pei DS. The biological function of IGF2BPs and their role in tumorigenesis. Invest New Drugs. 2021;39:1682–93. 34251559 10.1007/s10637-021-01148-9

[26] Ramesh-Kumar D, Guil S. The IGF2BP family of RNA binding proteins links epitranscriptomics to cancer. Semin Cancer Biol. 2022;86:18–31. 35643219 10.1016/j.semcancer.2022.05.009

[27] Wang G, Huang Z, Liu X, Huang W, Chen S, Zhou Y, Li D, Singer RH, Gu W. IMP1 suppresses breast tumor growth and metastasis through the regulation of its target mRNAs. Oncotarget. 2016;7:15690–702. 26910917 10.18632/oncotarget.7464 PMC4941270

[28] Kessler SM, Lederer E, Laggai S, Golob-Schwarzl N, Hosseini K, Petzold J, Schweiger C, Reihs R, Keil M, Hoffmann J, Mayr C, Kiesslich T, Pichler M, et al. IMP2/IGF2BP2 expression, but not IMP1 and IMP3, predicts poor outcome in patients and high tumor growth rate in xenograft models of gallbladder cancer. Oncotarget. 2017;8:89736–45. 29163784 10.18632/oncotarget.21116 PMC5685705

[29] Manieri NA, Drylewicz MR, Miyoshi H, Stappenbeck TS. Igf2bp1 is required for full induction of Ptgs2 mRNA in colonic mesenchymal stem cells in mice. Gastroenterology. 2012;143:110-21.e10. 22465430 10.1053/j.gastro.2012.03.037 PMC3383944

[30] Hamilton KE, Chatterji P, Lundsmith ET, Andres SF, Giroux V, Hicks PD, Noubissi FK, Spiegelman VS, Rustgi AK. Loss of stromal IMP1 promotes a tumorigenic microenvironment in the colon. Mol Cancer Res. 2015;13:1478–86. 26194191 10.1158/1541-7786.MCR-15-0224 PMC4644674

[31] Peng Y, Zhang Z, Yang G, Dai Z, Cai X, Liu Z, Yun Q, Xu L. N6-methyladenosine reader protein IGF2BP1 suppresses CD8 + T cells-mediated tumor cytotoxicity and apoptosis in colon cancer. Apoptosis. 2024;29:331–43. 37848671 10.1007/s10495-023-01893-7

[32] Ni Z, Sun P, Zheng J, Wu M, Yang C, Cheng M, Yin M, Cui C, Wang G, Yuan L, Gao Q, Li Y. JNK signaling promotes bladder cancer immune escape by regulating METTL3-mediated m6A modification of PD-L1 mRNA. Cancer Res. 2022;82:1789–802. 35502544 10.1158/0008-5472.CAN-21-1323

[33] Deng H, Yao H, Zhou S, He C, Huang Y, Li Y, Chen H, Shu J. Pancancer analysis uncovers an immunological role and prognostic value of the m6A reader IGF2BP2 in pancreatic cancer. Mol Cell Probes. 2024;73: 101948. 38122949 10.1016/j.mcp.2023.101948

[34] Xie J, Huang Z, Jiang P, Wu R, Jiang H, Luo C, Hong H, Yin H. Elevated N6-methyladenosine RNA levels in peripheral blood immune cells: a novel predictive biomarker and therapeutic target for colorectal cancer. Front Immunol. 2021;12: 760747. 34659267 10.3389/fimmu.2021.760747 PMC8515146

[35] Jeltsch A, Jurkowska RZ. Allosteric control of mammalian DNA methyltransferases—a new regulatory paradigm. Nucleic Acids Res. 2016;44:8556–75. 27521372 10.1093/nar/gkw723 PMC5062992

[36] Ehrlich M, Lacey M. DNA methylation and differentiation: silencing, upregulation and modulation of gene expression. Epigenomics. 2013;5:553–68. 24059801 10.2217/epi.13.43 PMC3864898

[37] Yu H, Guo Y, Chen J, Chen X, Jia P, Zhao Z. Rewired pathways and disrupted pathway crosstalk in Schizophrenia transcriptomes by multiple differential coexpression methods. Genes (Basel). 2021;12:665. 33946654 10.3390/genes12050665 PMC8146818

[38] Kim HJ, Ji YR, Lee YM. Crosstalk between angiogenesis and immune regulation in the tumor microenvironment. Arch Pharm Res. 2022;45:401–16. 35759090 10.1007/s12272-022-01389-z PMC9250479

[39] Cao Q, Chen Y. Integrated analyses of m(6)A regulator-mediated methylation modification patterns and tumor microenvironment infiltration characterization in pan-cancer. Int J Mol Sci. 2022;23:11182. 36232485 10.3390/ijms231911182 PMC9570346

[40] Wang J, Lu Q, Chen X, Aifantis I. Targeting MHC-I inhibitory pathways for cancer immunotherapy. Trends Immunol. 2024;45:177–87. 38433029 10.1016/j.it.2024.01.009 PMC12117934

[41] Rock KL, Reits E, Neefjes J. Present yourself! By MHC class I and MHC class II molecules. Trends Immunol. 2016;37:724–37. 27614798 10.1016/j.it.2016.08.010 PMC5159193

[42] Axelrod ML, Cook RS, Johnson DB, Balko JM. Biological consequences of MHC-II expression by tumor cells in cancer. Clin Cancer Res. 2019;25:2392–402. 30463850 10.1158/1078-0432.CCR-18-3200 PMC6467754

[43] Montauti E, Oh DY, Fong L. CD4(+) T cells in antitumor immunity. Trends Cancer. 2024;10:969–85. 39242276 10.1016/j.trecan.2024.07.009 PMC11464182

[44] Wu X, Li T, Jiang R, Yang X, Guo H, Yang R. Targeting MHC-I molecules for cancer: function, mechanism, and therapeutic prospects. Mol Cancer. 2023;22:194. 38041084 10.1186/s12943-023-01899-4 PMC10693139

[45] Wang Y, Lei H, Yan B, Zhang S, Xu B, Lin M, Shuai X, Huang J, Pang J. Tumor acidity-activatable macromolecule autophagy inhibitor and immune checkpoint blockade for robust treatment of prostate cancer. Acta Biomater. 2023;168:593–605. 37474083 10.1016/j.actbio.2023.07.018

[46] Kumar S, Chatterjee M, Ghosh P, Ganguly KK, Basu M, Ghosh MK. Targeting PD-1/PD-L1 in cancer immunotherapy: an effective strategy for treatment of triple-negative breast cancer (TNBC) patients. Genes Dis. 2023;10:1318–50. 37397537 10.1016/j.gendis.2022.07.024 PMC10311058

[47] Pietrantonio F, Loupakis F, Randon G, Raimondi A, Salati M, Trapani D, Pagani F, Depetris I, Maddalena G, Morano F, Corallo S, Prisciandaro M, Corti F, et al. Efficacy and safety of immune checkpoint inhibitors in patients with microsatellite instability-high end-stage cancers and poor performance status related to high disease burden. Oncologist. 2020;25:803–9. 32369650 10.1634/theoncologist.2020-0014 PMC7485362

[48] Sun P, Luan Y, Cai X, Liu Q, Ren P, Xin P, Yu Y, Song B, Wang Y, Chang H, Ma H, Chen Y. Predicting mechanism of immune response in microsatellite instability colorectal cancer. Heliyon. 2024;10: e28120. 38545192 10.1016/j.heliyon.2024.e28120 PMC10965513

[49] Müller S, Bley N, Glaß M, Busch B, Rousseau V, Misiak D, Fuchs T, Lederer M, Hüttelmaier S. IGF2BP1 enhances an aggressive tumor cell phenotype by impairing miRNA-directed downregulation of oncogenic factors. Nucleic Acids Res. 2018;46:6285–303. 29660014 10.1093/nar/gky229 PMC6158595

[50] Tang J, Wang S, Weng M, Guo Q, Ren L, He Y, Cui Z, Cong M, Qin M, Yu J, Su R, Li X. The IGF2BP3-COPS7B axis facilitates mRNA translation to drive colorectal cancer progression. Cancer Res. 2023;83:3593–610. 37560971 10.1158/0008-5472.CAN-23-0557

[51] Jing X, Han C, Li Q, Li F, Zhang J, Jiang Q, Zhao F, Guo C, Chen J, Jiang T, Wang X, Chen Y, Huang C. IGF2BP3-EGFR-AKT axis promotes breast cancer MDA-MB-231 cell growth. Biochim Biophys Acta Mol Cell Res. 2023;1870: 119542. 37474008 10.1016/j.bbamcr.2023.119542

[52] Li M, Sun D, Song N, Chen X, Zhang X, Zheng W, Yu Y, Han C. Mutant p53 in head and neck squamous cell carcinoma: molecular mechanism of gain-of-function and targeting therapy (review). Oncol Rep. 2023;50:162. 37449494 10.3892/or.2023.8599 PMC10394732

[53] Panebianco F, Kelly LM, Liu P, Zhong S, Dacic S, Wang X, Singhi AD, Dhir R, Chiosea SI, Kuan SF, Bhargava R, Dabbs D, Trivedi S, et al. THADA fusion is a mechanism of IGF2BP3 activation and IGF1R signaling in thyroid cancer. Proc Natl Acad Sci USA. 2017;114:2307–12. 28193878 10.1073/pnas.1614265114 PMC5338560

[54] Shi R, Yu X, Wang Y, Sun J, Sun Q, Xia W, Dong G, Wang A, Gao Z, Jiang F, Xu L. Expression profile, clinical significance, and biological function of insulin-like growth factor 2 messenger RNA-binding proteins in non-small cell lung cancer. Tumour Biol. 2017;39:1010428317695928. 28381175 10.1177/1010428317695928

[55] Newell-Price J, Clark AJ, King P. DNA methylation and silencing of gene expression. Trends Endocrinol Metab. 2000;11:142–8. 10754536 10.1016/s1043-2760(00)00248-4

[56] Siegfried Z, Simon I. DNA methylation and gene expression. Wiley Interdiscip Rev Syst Biol Med. 2010;2:362–71. 20836034 10.1002/wsbm.64

[57] Lee HM, Saw AK, Morris VK, Napolitano S, Bristow C, Srinivasan S, Peoples M, Sorokin A, Kanikarla Marie P, Schulz J, Singh AK, Terranova C, Coker O, et al. Epigenome reprogramming through H3K27 and H3K4 trimethylation as a resistance mechanism to DNA methylation inhibition in BRAFV600E-mutated colorectal cancer. Clin Cancer Res. 2024;30:5166–79. 39269307 10.1158/1078-0432.CCR-24-1166 PMC11829253

[58] Huang J, Shao Y, Gu W. Function and clinical significance of N6-methyladenosine in digestive system tumours. Exp Hematol Oncol. 2021;10:40. 34246319 10.1186/s40164-021-00234-1 PMC8272376

[59] Oh J, Hwa C, Jang D, Shin S, Lee SJ, Kim J, Lee SE, Jung HR, Oh Y, Jang G, Kwon O, An JY, Cho SY. Augmentation of the RNA m6A reader signature is associated with poor survival by enhancing cell proliferation and EMT across cancer types. Exp Mol Med. 2022;54:906–21. 35794212 10.1038/s12276-022-00795-z PMC9355997

[60] Busch B, Bley N, Müller S, Glaß M, Misiak D, Lederer M, Vetter M, Strauß HG, Thomssen C, Hüttelmaier S. The oncogenic triangle of HMGA2, LIN28B and IGF2BP1 antagonizes tumor-suppressive actions of the let-7 family. Nucleic Acids Res. 2016;44:3845–64. 26917013 10.1093/nar/gkw099 PMC4856984

[61] Elcheva IA, Gowda CP, Bogush D, Gornostaeva S, Fakhardo A, Sheth N, Kokolus KM, Sharma A, Dovat S, Uzun Y, Schell TD, Spiegelman VS. IGF2BP family of RNA-binding proteins regulate innate and adaptive immune responses in cancer cells and tumor microenvironment. Front Immunol. 2023;14:1224516. 37503349 10.3389/fimmu.2023.1224516 PMC10369348

[62] Wan W, Ao X, Chen Q, Yu Y, Ao L, Xing W, Guo W, Wu X, Pu C, Hu X, Li Z, Yao M, Luo D, et al. METTL3/IGF2BP3 axis inhibits tumor immune surveillance by upregulating N(6)-methyladenosine modification of PD-L1 mRNA in breast cancer. Mol Cancer. 2022;21:60. 35197058 10.1186/s12943-021-01447-y PMC8864846

[63] Li Z, Jia Y, Zhu H, Yuan H, Xing X, Xin Y, Ma T, Pang F, Zhang Y, Hu Y, Jia S, Ji J. Genomic landscape of microsatellite instability in Chinese tumors: a comparison of Chinese and TCGA cohorts. Int J Cancer. 2022;151:1382–93. 35567574 10.1002/ijc.34119

[64] Mancarella C, Morrione A, Scotlandi K. Novel regulators of the IGF system in cancer. Biomolecules. 2021;11:273. 33673232 10.3390/biom11020273 PMC7918569

[65] Kim T, Havighurst T, Kim K, Albertini M, Xu YG, Spiegelman VS. Targeting insulin-like growth factor 2 mRNA-binding protein 1 (IGF2BP1) in metastatic melanoma to increase efficacy of BRAF(V600E) inhibitors. Mol Carcinog. 2018;57:678–83. 29369405 10.1002/mc.22786 PMC6408214

[66] Roberts PJ, Der CJ. Targeting the Raf-MEK-ERK mitogen-activated protein kinase cascade for the treatment of cancer. Oncogene. 2007;26:3291–310. 17496923 10.1038/sj.onc.1210422

[67] Lu W, Yang S. METTL3/IGF2BP1 promotes the development of triple-negative breast cancer by mediating m6A methylation modification of PRMT7. Tissue Cell. 2024;93: 102690. 39709713 10.1016/j.tice.2024.102690

[68] Yang Y, Wu J, Liu F, He J, Wu F, Chen J, Jiang Z. IGF2BP1 promotes the liver cancer stem cell phenotype by regulating MGAT5 mRNA stability by m6A RNA methylation. Stem Cells Dev. 2021;30:1115–25. 34514861 10.1089/scd.2021.0153

[69] Belharazem D, Magdeburg J, Berton AK, Beissbarth L, Sauer C, Sticht C, Marx A, Hofheinz R, Post S, Kienle P, Ströbel P. Carcinoma of the colon and rectum with deregulation of insulin-like growth factor 2 signaling: clinical and molecular implications. J Gastroenterol. 2016;51:971–84. 26984550 10.1007/s00535-016-1181-5

[70] Silva Paiva R, Gomes I, Casimiro S, Fernandes I, Costa L. c-Met expression in renal cell carcinoma with bone metastases. J Bone Oncol. 2020;25: 100315. 33024658 10.1016/j.jbo.2020.100315 PMC7527574

[71] Zheng Y, Wang Y, Liu Y, Xie L, Ge J, Yu G, Zhao G. N6-methyladenosine modification of PTTG3P contributes to Colorectal cancer proliferation via YAP1. Front Oncol. 2021;11: 669731. 34660259 10.3389/fonc.2021.669731 PMC8515845

[72] Wang G, Zhou H, Gu Z, Gao Q, Shen G. Oct4 promotes cancer cell proliferation and migration and leads to poor prognosis associated with the survivin/STAT3 pathway in hepatocellular carcinoma. Oncol Rep. 2018;40:979–87. 29901157 10.3892/or.2018.6491

