# Supplementary material for: A pan-cancer analysis reveals the oncogenic and immunological role of insulin-like growth factor 2 mRNA-binding protein family members
Source: Discov Oncol. 2025 Mar 15;16:323. doi: 10.1007/s12672-025-02077-2 (PMC11910485; doi:10.1007/s12672-025-02077-2)
Supplement: Supplementary file 1 — Additional file 1. [file 12672_2025_2077_MOESM1_ESM.docx]

**A pan-cancer analysis reveals the oncogenic and immunological role of insulin-like growth factor 2 mRNA-binding protein family members**

Fuling Zeng ^1,^ , Liuyan Chen ^2,^ , Jing Li ^2,^ , Wenna Yu ^3,^ , Niya Sa ^2,^ , Keke Zhang ^2,^ , Chen Qu ^2**,^ , Daolin Wen^1*,^

1 Department of Laboratory Medicine, Shenzhen Guangming District People's Hospital, Shenzhen, Guangdong, 518000, China.

2 Department of Pathophysiology, School of Medicine, Jinan University, Guangzhou, Guangdong 510632, China.

3 College of Pharmacy, Jinan University, Guangzhou, Guangdong, 510632, China.

* Corresponding Author: Daolin Wen; Email: [wdl70@163.com](mailto:wdl70@163.com,)

** Corresponding Author: Chen Qu; Email: [chenqu@jnu.edu.cn](mailto:chenqu@jnu.edu.cn)

**Supplementary materials**

**Supplementary Figures**

**Supplementary Figure 1. Univariate Cox regression analysis of IGF2BP genes family.**

**
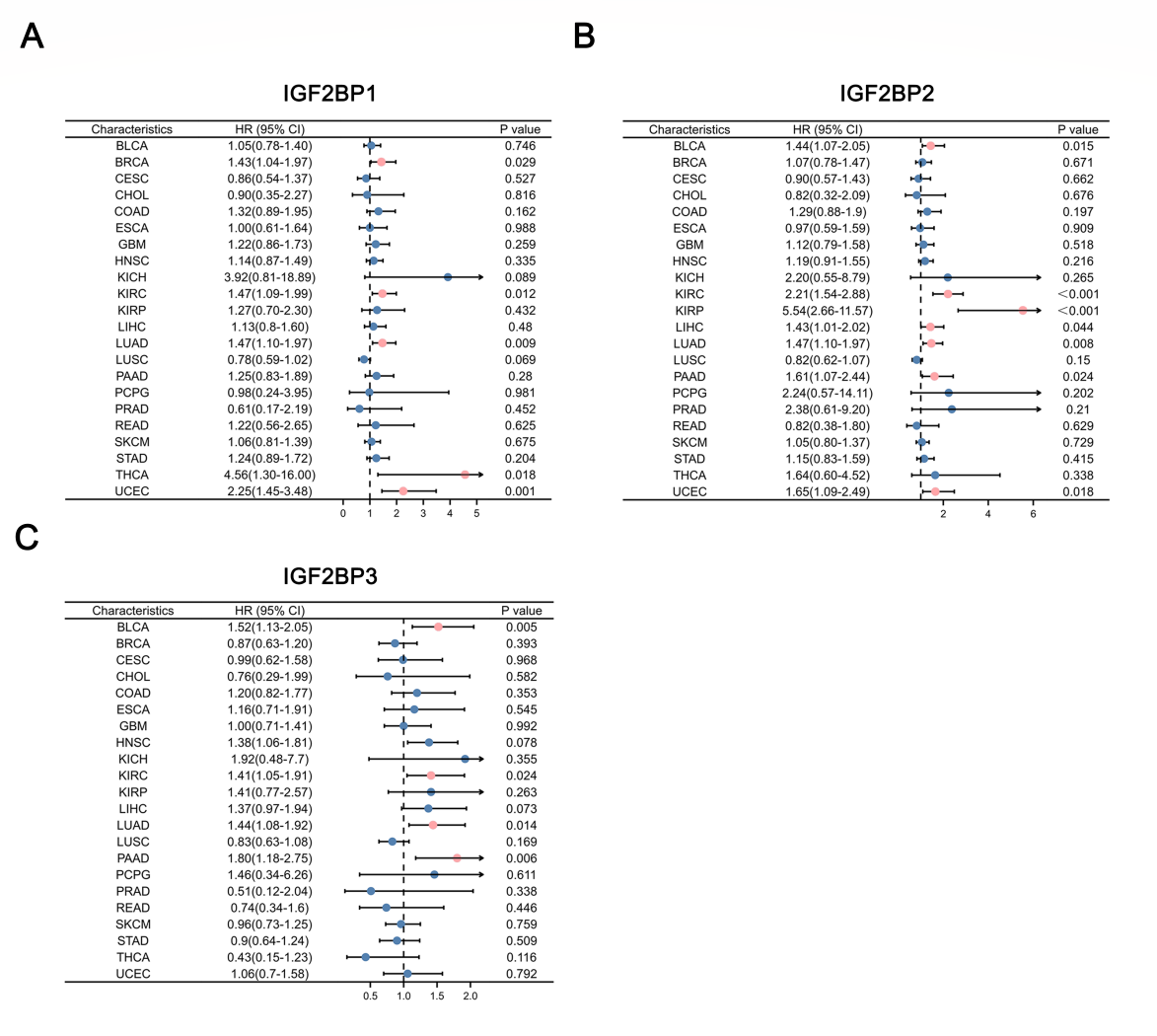
**

**A-C** Forest plots show hazard ratio (HR) and 95% confidence interval (CI) and p-value for IGF2BP1/2/3 in different tumors.

**Supplementary Figure 2. Evaluation of the prognostic value of IGF2BP2 in LUSC.**

**
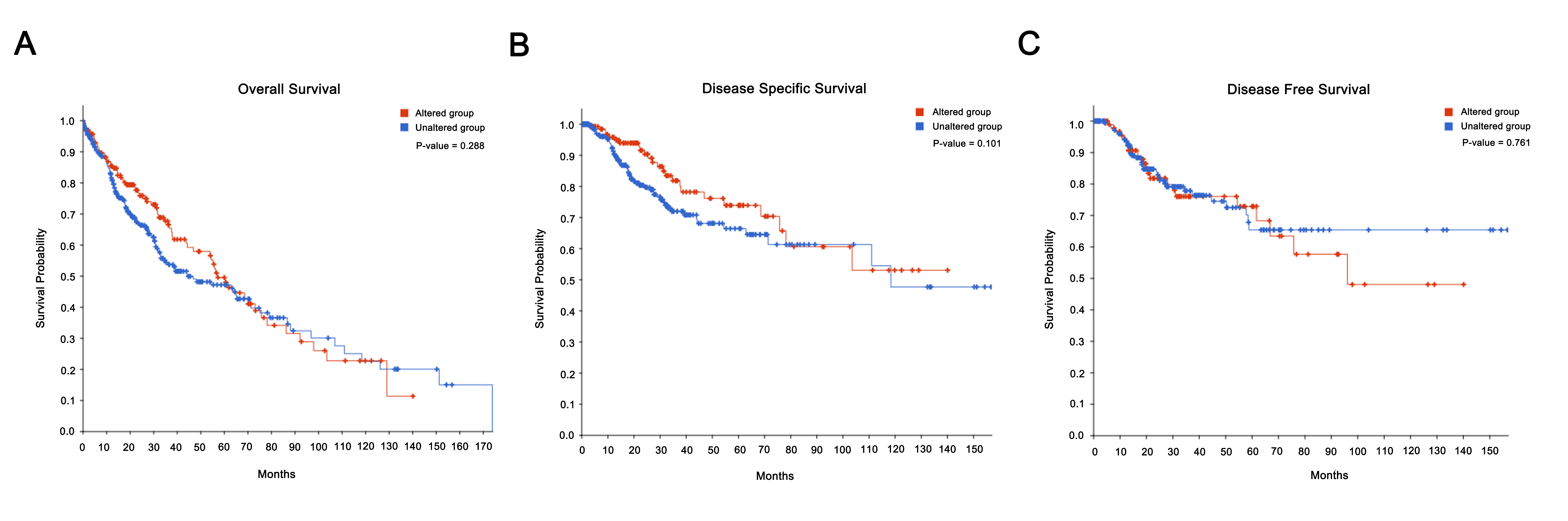
A-C** The prognostic survival curves between low and high IGF2BP1/2/3 expression subgroups.

**Supplementary Figure 3. The GO and KEGG enrichment analysis on co-expressed genes of IGF2BPs gene family.**

**
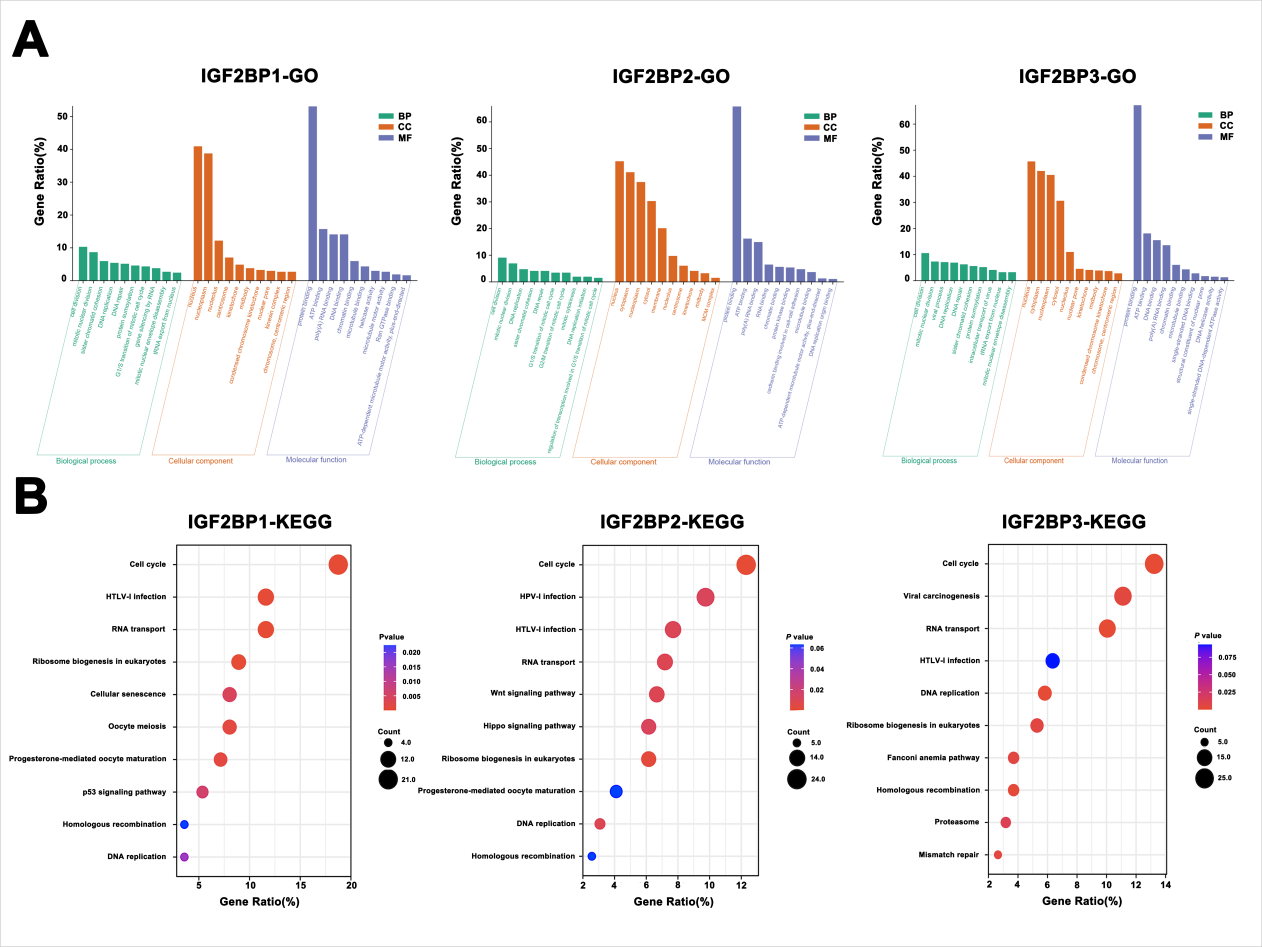
A** The GO enrichment analysis on co-expressed genes of IGF2BP1/2/3; **B** The KEGG enrichment analysis on co-expressed genes of IGF2BP1/2/3.

**Supplementary Tables**

**Supplementary Table 1.** **Abbreviation table**

| Acronyms |  | Full name |
| --- | --- | --- |
| IGF2BPs |  | Insulin-like growth factor 2 mRNA-binding proteins. |
| TCGA |  | The Cancer Genome Atlas |
| GEO |  | Gene Expression Omnibus |
| GDSC |  | Genomics of Drug Sensitivity in Cancer |
| CTRP |  | Cancer Therapeutics Response Portal |
| BLCA |  | Bladder Urothelial Carcinoma |
| BRCA |  | Breast invasive carcinoma |
| CESC |  | Cervical squamous cell carcinoma and endocervical adenocarcinoma |
| CHOL |  | Cholangio carcinoma |
| COAD |  | Colon adenocarcinoma |
| ESCA |  | Esophageal carcinoma |
| GBM |  | Glioblastoma multiforme |
| HNSC |  | Head and Neck squamous cell carcinoma |
| KICH |  | Kidney Chromophobe |
| KIRC |  | Kidney renal clear cell carcinoma |
| KIRP |  | Kidney renal papillary cell carcinoma |
| LIHC |  | Liver hepatocellular carcinoma |
| LUAD |  | Lung adenocarcinoma |
| LUSC |  | Lung squamous cell carcinoma |
| PAAD |  | Pancreatic adenocarcinoma |
| PCPG |  | Pheochromocytoma and Paraganglioma |
| PRAD |  | Prostate adenocarcinoma |
| READ |  | Rectum adenocarcinoma |
| SKCM |  | Skin Cutaneous Melanoma |
| STAD |  | Stomach adenocarcinoma |
| THCA |  | Thyroid carcinoma |
| UCEC |  | Uterine Corpus Endometrial Carcinoma |
| OS |  | Overall Survival |
| DFS |  | Disease-Free Survival |
| DSS |  | Disease-Specific Survival |
| HR |  | Hazard Ratio |
| SNV |  | Single Nucleotide Variations |
| CNV |  | Copy Number Variations |
| GO |  | Gene Ontology |
| KEGG |  | Kyoto Encyclopedia of Genes and Genomes |
| CR |  | Complete Response |
| PR |  | Partial Response |
| SD |  | Stable Disease |
| PD |  | Progressive Disease |
| EMT |  | Epithelial-Mesenchymal Transition |
| TME |  | Tumor Microenvironment |
| TMB |  | Tumor Mutation Burden |
| MSI |  | microsatellite instability |

**Supplementary Table 2. Sample distribution of RNA-seq data for each tumor**

| **Abbreviation** | **Full name** | **Tumor Samples** | **Normal Samples** |
| --- | --- | --- | --- |
| BLCA | Bladder Urothelial Carcinoma | 408 | 19 |
| BRCA | Breast invasive carcinoma | 1093 | 112 |
| CESC | Cervical squamous cell carcinoma and endocervical  adenocarcinoma | 304 | 3 |
| COAD | Colon adenocarcinoma | 457 | 41 |
| CHOL | Cholangio carcinoma | 36 | 9 |
| ESCA | Esophageal carcinoma | 184 | 11 |
| GBM | Glioblastoma multiforme | 153 | 5 |
| HNSC | Head and Neck squamous cell carcinoma | 520 | 44 |
| KICH | Kidney Chromophobe | 66 | 25 |
| KIRC | Kidney renal clear cell carcinoma | 533 | 72 |
| KIRP | Kidney renal papillary cell carcinoma | 290 | 32 |
| LIHC | Liver hepatocellular carcinoma | 371 | 50 |
| LUAD | Lung adenocarcinoma | 515 | 59 |
| LUSC | Lung squamous cell carcinoma | 501 | 51 |
| PAAD | Pancreatic adenocarcinoma | 178 | 4 |
| PCPG | Pheochromocytoma and Paraganglioma | 179 | 3 |
| PRAD | Prostate adenocarcinoma | 497 | 52 |
| READ | Rectum adenocarcinoma | 166 | 10 |
| SKCM | Skin Cutaneous Melanoma | 103 | 0 |
| STAD | Stomach adenocarcinoma | 415 | 35 |
| THCA | Thyroid carcinoma | 501 | 59 |
| UCEC | Uterine Corpus Endometrial Carcinoma | 545 | 35 |

**Supplementary Table 3. Sample distribution of survival data for each tumor**

|  | Survival data | | |
| --- | --- | --- | --- |
|  | N (OS) | N (DFS) | N (DSS) |
| BLCA | 412 | 163 | 354 |
| BRCA | 1096 | 912 | 1028 |
| CESC | 307 | 171 | 290 |
| COAD | 478 | 182 | 420 |
| CHOL | 45 | 25 | 41 |
| ESCA | 185 | 73 | 159 |
| GBM | 592 | 3 | 548 |
| HNSC | 526 | 122 | 434 |
| KICH | 112 | 70 | 109 |
| KIRC | 537 | 112 | 470 |
| KIRP | 290 | 177 | 274 |
| LIHC | 376 | 282 | 324 |
| LUAD | 513 | 278 | 442 |
| LUSC | 498 | 252 | 372 |
| PAAD | 185 | 58 | 164 |
| PCPG | 179 | 159 | 177 |
| PRAD | 499 | 337 | 495 |
| READ | 169 | 47 | 169 |
| SKCM | 461 | 0 | 429 |
| STAD | 436 | 225 | 368 |
| THCA | 503 | 354 | 498 |
| UCEC | 547 | 403 | 516 |

**Supplementary Table 4. Sample distribution of the pathological stage data for each tumor**

|  | Pathological stage | | | |  |
| --- | --- | --- | --- | --- | --- |
|  | N (Stage I) | N (Stage II) | N (Stgae III) | N (Stage IV) | total |
| BLCA | 4 | 131 | 143 | 138 | 416 |
| BRCA | 183 | 621 | 244 | 18 | 1066 |
| COAD | 82 | 189 | 134 | 67 | 472 |
| CHOL | 19 | 14 | 4 | 10 | 47 |
| ESCA | 17 | 78 | 55 | 9 | 159 |
| KICH | 53 | 33 | 19 | 7 | 112 |
| KIRC | 276 | 59 | 125 | 83 | 543 |
| KIRP | 173 | 22 | 52 | 15 | 262 |
| LIHC | 177 | 88 | 86 | 5 | 356 |
| LUAD | 298 | 127 | 85 | 26 | 536 |
| LUSC | 246 | 163 | 85 | 7 | 501 |
| PAAD | 21 | 152 | 4 | 6 | 183 |
| READ | 33 | 51 | 52 | 24 | 160 |
| SKCM | 78 | 140 | 172 | 24 | 414 |
| STAD | 57 | 129 | 182 | 43 | 411 |
| THCA | 290 | 52 | 114 | 57 | 513 |

**Supplementary Table 5. Sample distribution of the gene mutation data for each tumor**

|  | Mutation data | | |
| --- | --- | --- | --- |
|  | N (gene alteration) | N (SNV) | N (CNV) |
| BLCA | 410 | 411 | 408 |
| BRCA | 1066 | 1026 | 1080 |
| CESC | 291 | 291 | 295 |
| COAD | 534 | 407 | 451 |
| CHOL | 36 | 36 | 36 |
| ESCA | 182 | 185 | 184 |
| GBM | 397 | 403 | 577 |
| HNSC | 515 | 509 | 522 |
| KICH | 65 | 66 | 66 |
| KIRC | 402 | 370 | 528 |
| KIRP | 276 | 282 | 288 |
| LIHC | 366 | 365 | 370 |
| LUAD | 566 | 567 | 516 |
| LUSC | 484 | 485 | 501 |
| PAAD | 179 | 178 | 184 |
| PCPG | 178 | 184 | 162 |
| PRAD | 494 | 36 | 492 |
| SKCM | 440 | 468 | 367 |
| STAD | 436 | 439 | 441 |
| THCA | 490 | 500 | 499 |
| UCEC | 517 | 531 | 539 |

**Supplementary Table 6. Sample distribution of the DNA methylation data for each tumor**

|  | DNA methylation | |  |
| --- | --- | --- | --- |
|  | N (Tumor) | N (Normal) | total |
| BLCA | 418 | 21 | 439 |
| BRCA | 793 | 97 | 890 |
| CESC | 307 | 3 | 310 |
| COAD | 313 | 37 | 350 |
| CHOL | 36 | 9 | 45 |
| ESCA | 185 | 16 | 201 |
| GBM | 140 | 2 | 142 |
| HNSC | 528 | 50 | 578 |
| KIRC | 324 | 160 | 484 |
| KIRP | 275 | 45 | 320 |
| LIHC | 377 | 50 | 427 |
| LUAD | 473 | 32 | 505 |
| LUSC | 370 | 42 | 412 |
| PAAD | 184 | 10 | 194 |
| PCPG | 179 | 3 | 182 |
| PRAD | 502 | 50 | 552 |
| READ | 98 | 7 | 105 |
| STAD | 395 | 2 | 397 |
| THCA | 507 | 56 | 563 |
| UCEC | 418 | 21 | 439 |

**Supplementary Table 7.** Relationship between IGF2BP2 expression and clinical parameters in LUSC.

| **Characteristics** | **LUSC** | | ***P* value** |
| --- | --- | --- | --- |
|  | **IGF2BP2** | |  |
|  | **Low** | **High** |  |
| n | 251 | 251 |  |
| **Age, n (%)** |  |  | **0.450** |
| <= 65 | 92 (18.7%) | 99 (20.1%) |  |
| > 65 | 156 (31.6%) | 146 (29.6%) |  |
| **Gender, n (%)** |  |  | **0.264** |
| Female | 71 (14.1%) | 60 (12%) |  |
| Male | 180 (35.9%) | 191 (38%) |  |
| **Race, n (%)** |  |  | **0.355** |
| Asian | 6 (1.5%) | 3 (0.8%) |  |
| Black or African American | 12 (3.1%) | 18 (4.6%) |  |
| White | 171 (44%) | 179 (46%) |  |
| **Pathologic stage, n (%)** |  |  | **0.353** |
| Stage I | 129 (25.9%) | 116 (23.3%) |  |
| Stage II | 72 (14.5%) | 90 (18.1%) |  |
| Stage III | 45 (9%) | 39 (7.8%) |  |
| Stage IV | 3 (0.6%) | 4 (0.8%) |  |
| **Pathologic T stage, n (%)** |  |  | **0.519** |
| T1 | 64 (12.7%) | 50 (10%) |  |
| T2 | 141 (28.1%) | 153 (30.5%) |  |
| T3 | 35 (7%) | 36 (7.2%) |  |
| T4 | 11 (2.2%) | 12 (2.4%) |  |
| **Pathologic N stage, n (%)** |  |  | **0.055** |
| N0 | 164 (33.1%) | 156 (31.5%) |  |
| N1 | 54 (10.9%) | 77 (15.5%) |  |
| N2 | 24 (4.8%) | 16 (3.2%) |  |
| N3 | 4 (0.8%) | 1 (0.2%) |  |
| **Pathologic M stage, n (%)** |  |  | **0.964** |
| M0 | 210 (50.1%) | 202 (48.2%) |  |
| M1 | 3 (0.7%) | 4 (1%) |  |
| **Primary therapy outcome, n (%)** |  |  | **0.207** |
| PD&SD | 20 (5.5%) | 28 (7.8%) |  |
| PR&CR | 161 (44.6%) | 152 (42.1%) |  |
| **OS event, n (%)** |  |  | **0.207** |
| Alive | 136 (27.1%) | 150 (29.9%) |  |
| Dead | 115 (22.9%) | 101 (20.1%) |  |
| **DSS event, n (%)** |  |  | **0.586** |
| No | 179 (39.8%) | 182 (40.4%) |  |
| Yes | 47 (10.4%) | 42 (9.3%) |  |
| **PFI event, n (%)** |  |  | **0.557** |
| No | 180 (35.9%) | 174 (34.7%) |  |
| Yes | 71 (14.1%) | 77 (15.3%) |  |

**Supplementary Table 8.** **IGF2BP1/2/3 respective interacting proteins**

| **Interacting Proteins** | | | | | | | | | | | |
| --- | --- | --- | --- | --- | --- | --- | --- | --- | --- | --- | --- |
| **IGF2BP1** | | | | **IGF2BP2** | | | | **IGF2BP3** | | | |
| **YBX1** | **EIF4A2** | **PLEKHA7** | **SAFB** | **HMGA2** | **ALKBH3** | **PRLR** | **SLC17A4** | **IGF2** | **SUPV3L1** | **HNRNPH2** | **RPS15** |
| **IGF2** | **CPSF1** | **CUL7** | **PUS1** | **IGF2** | **XRN2** | **IRS2** | **METTL17** | **IGF2BP1** | **FASTKD2** | **ZMYM1** | **HSPE1-MOB4** |
| **HNRNPU** | **TUT7** | **DAP3** | **PSPC1** | **FTO** | **LYPLAL1** | **PRDX1** | **MRPS2** | **HNRNPA2B1** | **TNPO3** | **CLEC14A** | **MYH14** |
| **MYC** | **MEPCE** | **ZNF607** | **IGFBP2** | **HHEX** | **ESM1** | **LSM14B** | **SMAD2** | **HMGA2** | **CPSF3** | **EGFR** | **ZNF746** |
| **SYNCRIP** | **CPSF7** | **CCDC8** | **NOP58** | **CDKAL1** | **CTAG2** | **CAPRIN1** | **DDX21** | **YTHDF1** | **NUF2** | **AFP** | **RPL11** |
| **DHX9** | **CEP250** | **ASB2** | **XRN1** | **DHX9** | **ILF2** | **MMUT** | **TM9SF3** | **ELAVL1** | **TNRC6C** | **YAP1** | **RPS17** |
| **IGF2BP3** | **CELF1** | **ARID3B** | **RRM1** | **HNRNPC** | **IMP3** | **GNPDA2** | **PCSK1** | **IGF2BP2** | **HNRNPK** | **LPP** | **CDK2** |
| **ELAVL1** | **IGFBP1** | **RECQL4** | **SEMA3A** | **HNRNPA2B1** | **CPSF6** | **IGF2R** | **C9orf92** | **YTHDF3** | **KHDRBS1** | **CBX8** | **KRAS** |
| **ACTB** | **CDK9** | **SLC25A16** | **RBM38** | **YTHDF1** | **HNRNPM** | **IGF1** | **UCP2** | **YTHDC1** | **RPS6** | **EIF3H** | **SRSF1** |
| **YTHDF1** | **PUM1** | **KHDRBS2** | **CDK6** | **YTHDC1** | **IGF1R** | **PTBP3** | **TSC1** | **YTHDF2** | **AURKA** | **DIRC1** | **CCND1** |
| **HNRNPA2B1** | **RPS2** | **SEC62** | **CDH2** | **YTHDF2** | **LSM14A** | **HINT1** | **PAX4** | **METTL3** | **NUFIP2** | **TMOD3** | **SNRPA** |
| **CSDE1** | **EIF3F** | **CLEC14A** | **ATXN2** | **YTHDF3** | **NKX6-3** | **HSPA6** | **DHX57** | **YTHDC2** | **MUCL1** | **PBK** | **POU5F1** |
| **PABPC1** | **MRPS22** | **CNOT1** | **ZDHHC19** | **VIRMA** | **ADAM30** | **CIT** | **EIF2AK2** | **RBMX** | **DHX9** | **NME1** | **EIF2AK2** |
| **BTRC** | **RBM39** | **TNRC6B** | **RBM42** | **SLC30A8** | **DGKB** | **LRRK2** | **SNAI1** | **METTL14** | **ECT2** | **TNRC6B** | **ARID3A** |
| **MATR3** | **R3HDM1** | **FUBP3** | **BDNF** | **METTL3** | **ALDH1A3** | **ANKHD1** | **KRAS** | **VIRMA** | **EIF4E** | **NEFL** | **NSUN2** |
| **YTHDC1** | **CPSF3** | **IVNS1ABP** | **PATL1** | **YTHDC2** | **IGFBP2** | **IFI16** | **EIF3C** | **FTO** | **RPS16** | **FOXK2** | **HNRNPAB** |
| **HNRNPC** | **TERF1** | **LARP4** | **HDGF** | **IGF2BP3** | **LAMB1** | **FAM241A** | **PTPRD** | **HNRNPC** | **RTRAF** | **HNRNPA3** | **UBE2C** |
| **MATR3-2** | **MSI2** | **ELAVL3** | **MMUT** | **METTL14** | **MKRN1** | **FBXO45** | **TMEM18** | **YBX1** | **RPS3** | **LRIG1** | **RRM2** |
| **YTHDF2** | **FAM98A** | **MEX3C** | **HBE1** | **IGF2BP1** | **DDX53** | **ZC3H7A** | **MAEA** | **LIN28A** | **INS-IGF2** | **ABCA1** | **AKR1B1** |
| **YTHDF3** | **ANLN** | **OBSL1** | **SGPP2** | **TSPAN8** | **TIAL1** | **MTCH2** | **GSPT1** | **MATR3** | **GDF1** | **SLC25A16** | **CDCA5** |
| **G3BP1** | **RBMS2** | **GLCE** | **CDKN2A** | **KCNJ11** | **HMMR** | **NUP42** | **MSANTD1** | **ZC3H13** | **SKA3** | **MAPT** | **PSPC1** |
| **YTHDC2** | **CBX8** | **KIF18A** | **CCNA2** | **CDKN2B** | **CPSF7** | **HPGD** | **MSI2** | **ALKBH5** | **TRIM25** | **SLC25A5** | **DDX21** |
| **VIRMA** | **TNRC6C** | **SOD3** | **CCNB1** | **FAM120A** | **AGO4** | **PLEKHA7** | **ETV5** | **WTAP** | **IGF1R** | **RPL23** | **RNF43** |
| **ALKBH5** | **MMP10** | **BAG3** | **IGFBP3** | **RBMX** | **PRDX2** | **FXR2** | **FOXO3** | **RBM15B** | **CCL2** | **BANF1** | **FNDC3B** |
| **METTL3** | **SKP1** | **YWHAG** | **RBM8A** | **ELAVL1** | **PABPN1** | **PABPC4** | **DDX55** | **RBM15** | **FAM120A** | **MSI1** | **SNRPC** |
| **HMGA2** | **SRP54** | **DROSHA** | **HAS2** | **KCNQ1** | **EXT2** | **CMTR1** | **G6PC2** | **KIF20A** | **NCOA6** | **SOX2** | **GRSF1** |
| **IGF2BP2** | **KIF23** | **DUX4** | **PRMT1** | **TCF7L2** | **CHUK** | **CHD3** | **EIF4ENIF1** | **LIN28B** | **IDH1** | **LRRK2** | **ABCF1** |
| **LIN28B** | **FOXM1** | **NTN1** | **YAP1** | **CDKN2A** | **SRSF3** | **RECQL4** | **TNS1** | **FUS** | **H1-10** | **POM121** | **PTEN** |
| **MAPK4** | **TIA1** | **KRAS** | **DTL** | **ALKBH5** | **YBX3** | **AADAT** | **SH2B1** | **PABPC1** | **HSP90AA1** | **RPS10** | **SART3** |
| **FMR1** | **TCF7L2** | **SND1** | **CDK1** | **WTAP** | **LARP1** | **HSPA1L** | **MCRIP1** | **STAU1** | **PRC1** | **USP11** | **EIF3C** |
| **RBMX** | **NUDT21** | **TNKS2** | **PUM3** | **ZC3H13** | **HAS2** | **TRA2A** | **SOX4** | **ILF2** | **AGO4** | **TUBB2A** | **DCBLD2** |
| **METTL14** | **DGCR8** | **LAMTOR5** | **MEX3B** | **YBX1** | **TNRC6C** | **KIAA1211L** | **ZNF217** | **TTK** | **CAPRIN1** | **RPS18** | **GTSF1** |
| **WTAP** | **AXIN1** | **LRRK2** | **EIF4B** | **RBM15B** | **NUDT21** | **PUM1** | **HSCB** | **ELAVL2** | **WT1** | **ACTG1** | **CDC45** |
| **ZC3H13** | **CLSPN** | **MCU** | **PHOSPHO1** | **SOX2** | **CTAG1B** | **ABCC8** | **SLC2A4** | **MYC** | **AGO2** | **MYO1C** | **CWH43** |
| **GLI1** | **NEK2** | **LRRC59** | **NFKB1** | **RBM15** | **GLIS3** | **RBFOX2** | **PENK** | **METTL16** | **DDX6** | **MAP1B** | **MYCN** |
| **ELAVL4** | **KIF20A** | **WNT9A** | **PRDX1** | **EGFR** | **PAIP1** | **TRIM28** | **TMEM158** | **G3BP2** | **PRRC2A** | **MDH2** | **MELK** |
| **LRPPRC** | **NANOG** | **TRA2A** | **CCK** | **ELAVL2** | **RNF6** | **RRP1B** | **CPEB1** | **G3BP1** | **IGFBP1** | **TUBA1B** | **RBM14** |
| **STAU1** | **LARP1** | **EIF3B** | **GDF6** | **METTL16** | **LYRM1** | **SERBP1** | **CDK6** | **HNRNPU** | **TRUB2** | **MYL6** | **LARP1B** |
| **CTNNB1** | **IGF1R** | **LSM14A** | **HNRNPA0** | **FMR1** | **MAGEA3** | **RYBP** | **SCN9A** | **HMGA1** | **YBX3** | **CENPF** | **SOCS2** |
| **LIN28A** | **MCRIP2** | **SALL4** | **RPL5** | **JAZF1** | **BMI1** | **NLRP7** | **STRAP** | **DDX5** | **STXBP5** | **WWOX** | **GCSH** |
| **RBM15** | **ZCCHC3** | **ZMYM1** | **ANHX** | **CDC123** | **CBX8** | **SPRED2** | **SCN7A** | **HNRNPM** | **CAVIN1** | **RPS7** | **IGF2R** |
| **METTL16** | **SOX2** | **HDAC5** | **IFITM1** | **MTNR1B** | **STXBP5** | **UBE3A** | **GRB10** | **MKRN1** | **BMI1** | **CCDC8** | **TIAL1** |
| **STAU2** | **PHB** | **MPHOSPH6** | **BAIAP2L1** | **IRS1** | **PUM2** | **MIB1** | **ALX4** | **LY6K** | **NEIL3** | **RPL10A** | **PTBP3** |
| **MOV10** | **LSM14B** | **GAS1** | **DCST1** | **G3BP1** | **CPSF3** | **SLC16A11** | **FBL** | **PDK4** | **DDX3X** | **POM121C** | **NCBP1** |
| **DDX3X** | **PCSK4** | **BIN1** | **GSK3B** | **WFS1** | **DUSP9** | **HEXIM1** | **MFAP3L** | **USP10** | **ARHGEF4** | **TRIM28** | **ZC3HAV1** |
| **FTO** | **ZBTB17** | **SFPQ** | **CDC45** | **PABPC1** | **KLF14** | **PELO** | **MBNL3** | **HDGF** | **FOXM1** | **USP14** | **KIF11** |
| **RBM15B** | **RO60** | **CHD3** | **ADAMTS10** | **PTBP1** | **LARP6** | **PCIF1** | **ACLY** | **CDK6** | **CPSF2** | **CALM3** | **E2F7** |
| **HNRNPD** | **IPO13** | **H2BC3** | **MBNL1** | **HNRNPD** | **BTF3** | **SOCS2** | **INSRR** | **NUDT21** | **ITGB1** | **EXO1** | **PTTG1** |
| **PTBP1** | **MRPS28** | **HNRNPH2** | **EXO1** | **ZBED3** | **CPSF1** | **NR2C2** | **MYCN** | **LRPPRC** | **CPSF1** | **PCIF1** | **ZNF217** |
| **FXR2** | **EIF4A3** | **ZC3H18** | **FOXK1** | **RC3H1** | **ZMYM1** | **XRN1** | **SIRT1** | **SPOP** | **EIF3B** | **LHFPL6** | **TIA1** |
| **CPEB1** | **PARP12** | **NPM1** | **ADAM19** | **HMGA1** | **TRIM25** | **LARP4** | **TMBIM1** | **HNRNPH1** | **ERH** | **BRD7** | **LSM14A** |
| **FXR1** | **NSUN2** | **ANXA4** | **STAT3** | **PCBP1** | **CYP11B2** | **ZCCHC3** | **CIAO1** | **CBLL1** | **IMPDH2** | **COX6C** | **BAIAP2L1** |
| **HNRNPR** | **PTPRA** | **MBNL3** | **GEMIN5** | **EZH2** | **ACY1** | **PLEKHA4** | **FEN1** | **AGO1** | **ILF3** | **RPLP2** | **CCNA2** |
| **AGO2** | **PABPC4** | **RBMY1A1** | **RBPMS2** | **HNF1B** | **FUBP3** | **RBM45** | **GSPT2** | **CD44** | **PHB** | **TPX2** | **MRPS23** |
| **UPF1** | **CPSF4** | **GAP43** | **TRMT61B** | **HNRNPK** | **KLHDC9** | **CCDC8** | **ADAM19** | **LARP7** | **SNRPE** | **BUB1** | **EWSR1** |
| **PTEN** | **MRPS27** | **SRC** | **LARP4B** | **RBM3** | **CELF1** | **KNTC1** | **MSI1** | **HNRNPA1** | **YWHAE** | **CHD3** | **PAIP1** |
| **AGO1** | **KIF14** | **AFP** | **NKAP** | **SRSF1** | **RBMS1** | **ZBTB18** | **E2F1** | **HNRNPL** | **BIRC5** | **IGFBP2** | **KIF4A** |
| **QKI** | **NOP56** | **TOP2A** | **RBM41** | **CAMK1D** | **SLC35D3** | **WWP2** | **EWSR1** | **MOV10** | **BTF3** | **RPS29** | **U2AF2** |
| **CAPRIN1** | **MYO5A** | **TYR** | **AMOTL1** | **G3BP2** | **HSPA1A** | **TRIM31** | **PLEK2** | **NPM1** | **FBXW7** | **C4A** | **FAM151B** |
| **CBLL1** | **DDX18** | **HEXIM1** | **PNMA2** | **CBLL1** | **ADCY5** | **BRD7** | **PLPP1** | **RC3H1** | **EIF4A3** | **HEXIM1** | **HNRNPUL2** |
| **RC3H1** | **PCIF1** | **TUSC3** | **PHLPP2** | **ADAMTS9** | **NPM1** | **HECTD1** | **CDK5** | **MATR3-2** | **EIF2S1** | **KIF2C** | **BRCA2** |
| **HNRNPA1** | **SRSF10** | **USP14** | **SNRPC** | **KIF11** | **GPC3** | **NBEAL2** | **PEX5L** | **INS** | **KDR** | **TLE4** | **TRMT61A** |
| **ILF3** | **BIRC2** | **IFI16** | **LEF1** | **LIN28B** | **HNRNPDL** | **ZC3H18** | **NR6A1** | **FMR1** | **NCL** | **MYL12B** | **CCNE1** |
| **G3BP2** | **BTF3** | **RANBP1** | **IGFBPL1** | **STAU1** | **KIF23** | **WWOX** | **DROSHA** | **PTBP1** | **EEF1AKNMT** | **SLC25A11** | **DGCR8** |
| **PRRC2A** | **TIMELESS** | **ARID3A** | **BCL2** | **RALY** | **ANLN** | **ZNF746** | **HKDC1** | **RC3H2** | **SRSF7** | **LGALS3BP** | **SERPINB8** |
| **CPEB4** | **METTL4** | **REST** | **HIF1A** | **SYNCRIP** | **PHB** | **PDE10A** | **CENPW** | **HDGFL1** | **EIF4A1** | **TRA2A** | **IGF1** |
| **FUBP1** | **METAP2** | **KHDRBS1** | **CWH43** | **HNRNPH1** | **STAU2** | **ITPK1** | **SECISBP2** | **ANKRD17** | **PUM1** | **E2F1** | **MMUT** |
| **HNRNPDL** | **GSPT2** | **RRM2** | **LARP6** | **MYC** | **DDX6** | **UBE2E2** | **HK2** | **LRRC59** | **NANOG** | **STMN3** | **HBE1** |
| **PCBP2** | **LYPD1** | **TET1** | **DPPA4** | **LRPPRC** | **EEF1AKNMT** | **BCL11A** | **MCRIP2** | **MARCKS** | **CPSF4** | **DBT** | **MAD2L1** |
| **FUS** | **RTL10** | **EIF4G1** | **GRK5** | **NNMT** | **CPSF4** | **TARDBP** | **MAT2B** | **QKI** | **GAPDH** | **CCK** | **GSK3B** |
| **HNRNPK** | **VRK1** | **NXF1** | **GTSF1** | **ZFAND6** | **GIPR** | **CILP2** | **SRSF7** | **GPC3** | **TMPO** | **TOP2A** | **DLGAP5** |
| **HNRNPAB** | **DNMT3B** | **NONO** | **KLHL20** | **ILF3** | **FXR1** | **YBX2** | **SEC62** | **MKRN2** | **ZC3H7B** | **AKIRIN2** | **MRPL13** |
| **KIF11** | **CDKN1A** | **EEF1AKNMT** | **XPO5** | **LARP7** | **HNRNPAB** | **SMOX** | **ADIPOQ** | **RBM47** | **DHX57** | **PRDX1** | **RDM1** |
| **ALKBH1** | **CIT** | **BRD7** | **RBMS1** | **DDX3X** | **ADAM9** | **ASB2** | **TAS2R50** | **RBM3** | **SRSF3** | **MVP** | **AXL** |
| **PTBP2** | **TDRKH** | **TRMT112** | **TNFRSF21** | **MATR3-2** | **NDUFS3** | **SSR3** | **TRMT112** | **CTNNB1** | **CIT** | **ARPC1B** | **SLC2A1** |
| **AGO4** | **MKRN2** | **NR2C2** | **SRP68** | **CAVIN1** | **CPSF2** | **KIF18A** | **RETN** | **HNRNPD** | **HNRNPDL** | **ACTBL2** | **SEZ6L** |
| **LARP7** | **UBE2S** | **RDM1** | **DDX21** | **MATR3** | **INSR** | **IGFBP1** | **CDH1** | **SNRPB** | **CCT8** | **NR2C2** | **RBM42** |
| **CPEB3** | **PCBP1** | **BMI1** | **PEG10** | **GCKR** | **CTNNB1** | **MEX3A** | **KIF1B** | **CD274** | **RPS9** | **RPS23** | **CCNB2** |
| **MKRN1** | **SINHCAF** | **PAGE4** | **APOH** | **HNRNPU** | **CPEB4** | **SLC25A16** | **CDK2** | **PIH1D2** | **UPF1** | **RNF123** | **UPF2** |
| **CPEB2** | **POU5F1** | **AKAP10** | **DAZAP1** | **AGO2** | **ANK1** | **ACTB** | **CCNL1** | **EEF2** | **BRD4** | **RPL14** | **PDGFC** |
| **CD44** | **MRPS7** | **KCTD10** | **F2** | **CAPN10** | **ATF4** | **LAMTOR5** | **SLC2A2** | **PCSK4** | **CELF1** | **OTUD4** | **TRIP13** |
| **INS** | **IMPDH2** | **NEK4** | **ADAR** | **RC3H2** | **ECT2** | **SRSF10** | **OSBP2** | **ANLN** | **PRLR** | **RPL30** | **OIP5** |
| **DDX6** | **ODC1** | **AKIRIN2** | **CD274** | **HNRNPL** | **MC4R** | **PHLPP2** | **BRCA2** | **CDK1** | **KHDRBS2** | **CUL7** | **HELLS** |
| **HNRNPF** | **CROCC** | **PLEKHA4** | **WDR4** | **PRRC2A** | **LGR5** | **DGKG** | **CD274** | **RPL6** | **DCLK1** | **POTEE** | **BRCA1** |
| **TRIM71** | **MYCN** | **RBM45** | **LHPP** | **PPARG** | **PRDX3** | **SLC25A37** | **KCNK16** | **CDYL** | **TP73** | **RPL18** | **CERS6** |
| **DICER1** | **EIF4E** | **CHMP4C** | **EFTUD2** | **AGO1** | **HMG20A** | **CELF2** | **HNRNPUL2** | **KIF23** | **METTL4** | **NACA** | **CPEB1** |
| **RC3H2** | **EIF3H** | **ITFG1** | **AHSG** | **RAB5A** | **KIF20A** | **ENPP1** | **VAPA** | **CPSF6** | **CBX6** | **CLTC** | **SNAI1** |
| **FAM120A** | **TMPO** | **WWP2** | **TRMT6** | **C2CD4A** | **SLC2A1** | **UBE2O** | **ZFP36** | **PABPC4** | **PUM2** | **AP2B1** | **TMBIM6** |
| **SRSF1** | **CALN1** | **IFIT1** | **PTCD3** | **ARAP1** | **FITM1** | **HDGFL1** | **CDC6** | **RACK1** | **SRP14** | **SND1** | **CDKN2A** |
| **TIAL1** | **EZH2** | **MTF1** | **TSC1** | **ALKBH1** | **SP6** | **GRB14** | **POU5F1** | **KIF14** | **XRCC6** | **GSN** | **C1QBP** |
| **ALKBH3** | **TRIM28** | **TLE4** | **MTCH2** | **HIF1A** | **RBPMS** | **PROX1** | **IGFBP6** | **SYNCRIP** | **IGFBP3** | **SRSF10** | **CDK4** |
| **ZCCHC4** | **PRC1** | **TRIM31** | **PRRC2C** | **LIN28A** | **GAPDH** | **FUS** | **PABPN1L** | **ZCCHC4** | **WASF2** | **GRN** | **DTL** |
| **CCND1** | **FBXO32** | **NFX1** | **PIWIL4** | **INS-IGF2** | **HSPA1B** | **BAIAP2L1** | **CSDE1** | **DEPDC1** | **PTCD1** | **AKAP10** | **EIF1AX** |
| **MITF** | **TRIM25** | **HECTD1** | **ZNF217** | **QKI** | **MOV10** | **SPRY2** | **DNMT3A** | **AGT** | **YWHAQ** | **KCTD10** | **NANOS1** |
| **PUM2** | **EIF4G2** | **CBX6** | **CFL2** | **PCSK4** | **AKT1** | **CCND1** | **AFF4** | **PTGDR** | **CDKN1A** | **NEK4** | **DROSHA** |
| **HNRNPA3** | **SNRPA** | **SATB1** | **PRKN** | **MTOR** | **TUT4** | **PTEN** | **HNRNPH2** | **METTL5** | **CKAP5** | **RPL8** | **PABPC1L** |
| **MEX3A** | **AKT1** | **WWOX** | **TNF** | **NRARP** | **DGCR8** | **UCP1** | **APOOL** | **RPL37A** | **AKT1** | **PLEKHA4** | **RBMS2** |
| **YBX3** | **RBM47** | **ZNF746** | **BRCA1** | **ZCCHC4** | **VWA5A** | **U2AF2** | **PKM** | **ALKBH1** | **RPS2** | **CNTNAP1** | **DQX1** |
| **TP53** | **UPF2** | **CDK2** | **PBK** | **E2F6** | **EIF3H** | **EIF3B** | **NEGR1** | **PUF60** | **OBSL1** | **RPS11** | **MRPL15** |
| **HNRNPM** | **ZFP36** | **DDX1** | **CFL1** | **LARP4B** | **PA2G4** | **IGFBP3** | **MMP9** | **MEPCE** | **CYP1B1** | **MSI2** | **MTERF3** |
| **USP10** | **BCL9L** | **HNRNPH1** | **NSUN5** | **INS** | **RNF4** | **YAP1** | **FITM2** | **HNRNPF** | **PHGDH** | **RBM45** | **KCNF1** |
| **PABPN1** | **TARDBP** | **ZEB1** | **A1CF** | **HDGF** | **MRPL58** | **HNF1A** | **R3HDM1** | **RBMS1** | **MMP12** | **SPRTN** | **BRIX1** |
| **TUT4** | **KHSRP** | **MEIOC** | **SOX4** | **LDHA** | **PRC1** | **CWH43** | **MKRN2** | **TP53** | **CTCF** | **CHMP4C** | **CD8A** |
| **METTL5** | **EIF3C** | **LARP1B** | **PRDX2** | **CTCF** | **RBM39** | **ATXN2** | **CDH2** | **NFATC3** | **RPL7A** | **DYRK1A** | **PDCD6** |
| **AGT** | **MSI1** | **HOXB9** | **TRMT61A** | **AURKA** | **KIF14** | **PABPC5** | **NRAS** | **ACTB** | **RAE1** | **RPL21** | **HIPK3** |
| **XRN2** | **GAPDH** | **GRB2** | **MRPS10** | **BTRC** | **SHBG** | **RBM41** | **PKN2** | **KIF18A** | **DDX17** | **RPS3A** | **MRPL9** |
| **L1TD1** | **WNT16** | **EIF4ENIF1** | **SIRT1** | **HNRNPF** | **CCK** | **NANOG** | **NKAP** | **RPS20** | **RBM39** | **RPL22** | **MPHOSPH6** |
| **U2AF2** | **AURKA** | **MAT2A** | **FZD10** | **USP10** | **EIF4A3** | **GCK** | **SNRNP70** | **ARF6** | **RPL13** | **ZBTB18** | **SEC62** |
| **RPH3AL** | **TRIB1** | **IGDCC3** | **MKI67** | **CNOT1** | **MRPS34** | **GRSF1** | **ZCCHC9** | **KIF20B** | **FXR1** | **AP2A1** | **CHEK1** |
| **HNF4A** | **NCBP3** | **KCNG1** | **YBX2** | **AKAP12** | **NSUN2** | **ARID3A** | **ARG2** | **ALYREF** | **ENO1** | **CMTR1** | **CDCA7** |
| **CDH1** | **CHD4** | **STRBP** | **BRCA2** | **FHL2** | **FOXM1** | **ZEB1** | **CDCP1** | **FLT1** | **ITLN1** | **RPS4X** | **NCAPG** |
| **CELF2** | **SOCS2** | **E2F1** | **PENK** | **NOTCH2** | **CBX6** | **R3HDML** | **SMAD3** | **VTCN1** | **NRAS** | **TRIM31** | **TRMT6** |
| **EIF4A1** | **MRPS34** | **SNAI1** | **ALYREF** | **METTL5** | **THADA** | **MYOD1** | **NOP2** | **KPNA2** | **MRPS34** | **HECTD4** | **RTCB** |
| **ILF2** | **SRSF7** | **PTBP3** | **DNMT3A** | **TNF** | **ZFAND3** | **DDX1** | **INSIG2** | **DICER1** | **UCHL1** | **NFX1** | **SRP68** |
| **SERBP1** | **ECT2** | **RIDA** | **GDF3** | **DDX11** | **UPF1** | **IPO13** | **FAM120C** | **CNOT1** | **RFC4** | **RPS8** | **LARP6** |
| **CYFIP2** | **SSB** | **BIRC5** | **TGFBR2** | **RBM47** | **TP53** | **RIDA** | **ADRA2A** | **NONO** | **TUBB** | **HECTD1** | **SFPQ** |
| **HMGA1** | **DND1** | **RBM14** | **HELLS** | **C2CD4B** | **KEAP1** | **EIF4G2** | **NEUROD1** | **ASPM** | **FXR2** | **RPL31** | **WDHD1** |
| **SRSF3** | **GRSF1** | **NCL** | **RBM4** | **DICER1** | **PCBP2** | **MASTL** | **OR13D1** | **EIF2S3** | **EZH2** | **ARPC4** | **RPUSD4** |
| **CPSF2** | **FAM107B** | **SQSTM1** | **ZP4** | **DDX5** | **DCTN1** | **PARP12** | **CDK1** | **ALKBH3** | **APOBEC3D** | **EEF1D** | **HAS2** |
| **HOXA9** | **DDX5** | **TNRC6A** | **MAGEA4** | **TP53INP1** | **S100A10** | **KCNF1** | **STAT3** | **PCBP1** | **RPSA** | **RAB3GAP1** | **NEK2** |
| **ELAVL2** | **TOPBP1** | **SNRPB** | **BNIP3** | **ADAL** | **CLEC14A** | **PATL1** | **TMEM163** | **CPSF7** | **SMAD3** | **NBEAL2** | **MKI67** |
| **CPSF6** | **FASTK** | **RBFOX2** | **LSM12** | **MEPCE** | **TNRC6B** | **SLC26A9** | **NAPG** | **L1TD1** | **CCNB1** | **ZC3H18** | **MRPS18B** |
| **HNRNPL** | **ZFR** | **RICTOR** | **UHRF1** | **HNRNPA1** | **AGMO** | **TRA2B** | **GATA3** | **RCC1L** | **RECQL4** | **PLEKHA7** | **PCLAF** |
| **KCNF1** | **EGFR** | **MRPS23** | **CAMK2A** | **LAMC1** | **METTL4** | **SND1** | **KLHL42** | **RPL4** | **TUBB3** | **RPS13** | **GSPT2** |
| **CUL1** | **IGF2R** | **NOP2** | **DAZL** | **RBMS2** | **FBXW7** | **RO60** | **SNRPG** | **TOMM34** | **DNMT3B** | **DUX4** | **NGRN** |
